# A comparative summary of expression systems for the recombinant production of galactose oxidase

**DOI:** 10.1186/1475-2859-9-68

**Published:** 2010-09-13

**Authors:** Oliver Spadiut, Lisbeth Olsson, Harry Brumer

**Affiliations:** 1 Division of Glycoscience, School of Biotechnology, Royal Institute of Technology (KTH), SE-106 91 Stockholm, Sweden; 2 Industrial Biotechnology, Department of Chemical and Biological Engineering, Chalmers University of Technology, SE-412 96 Gothenburg, Sweden; 3 Wallenberg Wood Science Center, Royal Institute of Technology (KTH), SE-100 44 Stockholm, Sweden; 4 Wallenberg Wood Science Center, Chalmers University of Technology, SE-412 96 Gothenburg, Sweden

## Abstract

**Background:**

The microbes *Escherichia coli *and *Pichia pastoris *are convenient prokaryotic and eukaryotic hosts, respectively, for the recombinant production of proteins at laboratory scales. A comparative study was performed to evaluate a range of constructs and process parameters for the heterologous intra- and extracellular expression of genes encoding the industrially relevant enzyme galactose 6-oxidase (EC 1.1.3.9) from the fungus *Fusarium graminearum*. In particular, the wild-type *galox *gene from *F. graminearum*, an optimized variant for *E. coli *and a codon-optimized gene for *P. pastoris *were expressed without the native pro-sequence, but with a His-tag either at the N- or the C-terminus of the enzyme.

**Results:**

The intracellular expression of a codon-optimized gene with an N-terminal His_10_-tag in *E. coli*, using the pET16b^+ ^vector and BL21DE3 cells, resulted in a volumetric productivity of 180 U·L^-1^·h^-1^. The intracellular expression of the wild-type gene from *F. graminearum*, using the pPIC3.5 vector and the *P. pastoris *strain GS115, was poor, resulting in a volumetric productivity of 120 U·L^-1^·h^-1^. Furthermore, this system did not tolerate an N-terminal His_10_-tag, thus rendering isolation of the enzyme from the complicated mixture difficult. The highest volumetric productivity (610 U·L^-1^·h^-1^) was achieved when the wild-type gene from *F. graminearum *was expressed extracellularly in the *P. pastoris *strain SMD1168H using the pPICZα-system. A C-terminal His_6_-tag did not significantly affect the production of the enzyme, thus enabling simple purification by immobilized metal ion affinity chromatography. Notably, codon-optimisation of the *galox *gene for expression in *P. pastoris *did not result in a higher product yield (g protein·L^-1 ^culture). Effective activation of the enzyme to generate the active-site radical copper complex could be equally well achieved by addition of CuSO_4 _directly in the culture medium or post-harvest.

**Conclusions:**

The results indicate that intracellular production in *E. coli *and extracellular production in *P. pastoris *comprise a complementary pair of systems for the production of GalOx. The prokaryotic host is favored for high-throughput screening, for example in the development of improved enzymes, while the yeast system is ideal for production scale-up for enzyme applications.

## Background

Galactose 6-oxidase (GalOx; D-galactose:oxygen 6-oxidoreductase; EC 1.1.3.9) is a monomeric, free radical copper oxidase that oxidizes a range of primary alcohols to aldehydes, especially the C6 hydroxyl group of galactose [1]. GalOx is naturally secreted by a number of filamentous fungi, most prominently *Fusarium *spp. [2,3], with the GalOx from *F. graminearum *being perhaps the most well-studied representative [4-7]. Due to a particularly high selectivity for galactose and galactose-containing oligo- and polysaccharides, GalOx has been harnessed for a diversity of biotechnological applications. GalOx has been successfully applied in biosensors to measure the lactose concentration in dairy products [8,9] and the presence of glycoproteins in biomaterials [10]. It has also been used in chemical synthesis and various diagnostic applications in medicine [11,12]. Oxidation of galactosylated polysaccharides, in particular galactomannans and xyloglucans from plant seeds, has been employed for multivalent functionalization of these biopolymers [13-15]. The resulting aldehyde derivatives can be used directly e.g. as strength additives in the paper industry [14,16], oxidized to carboxylic acids [16] or further derivatized to install chemical functional groups [13].

**Figure 1:**
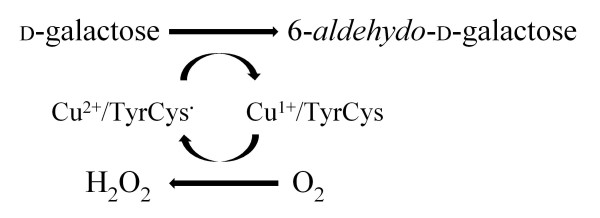
Catalytic reaction of GalOx

The catalytic two-electron oxidation catalyzed by GalOx follows a ping-pong bi-bi mechanism, mediated by a redox-active copper ion coordinated to a thioether-bridged tyrosylcysteine (Tyr-Cys) residue formed by post-translational oxidation [5,7]. In the fully oxidized (active) form of GalOx, the Tyr-Cys residue is in the radical form and the copper atom is in the +2 oxidation state (Cu^2+^/Tyr-Cys^**· **^form) [3,5]. Oxidation of primary alcohols to the corresponding aldehydes reduces the cofactor to the Cu^1+^/Tyr-Cys (non-radical) species, which is subsequently re-oxidized by O_2 _to the Cu^2+^/Tyr-Cys^**· **^form, yielding H_2_O_2 _as a product (Figure 1).

For fundamental studies and applications, the GalOx from *F. graminearum *has been recombinantly produced in a range of microbial hosts, including *Escherichia coli *[17,18], *Pichia pastoris *[5,14,19] and *Aspergillus nidulans *[20]. The factors dictating the choice of expression host are manifold, including growth rate, complexity of growth medium and handling, ease of transformation, potential for post-translational protein modification, ease of harvest, extent of endogenous contaminants, overall productivity, etc. In this context, *Escherichia coli *and *Pichia pastoris *have become the pre-eminent pro- and eukaryotic microbial hosts, respectively, for routine laboratory use. Despite individual reports of the successful use of *E. coli *and *P. pastoris *for GalOx production, a direct, practical comparison has not been performed thus far. In the present study, we have therefore explored a variety of codon-optimized and affinity-tagged variants of the *galox *gene from *F. graminearum *for the recombinant production of the enzyme in these hosts. This study highlights the advantages and limitations of particular gene construct-host systems, with respect to their suitability for high-throughput screening and up-scaling for enzyme applications.

## Materials and methods

### Genes and vectors

The nucleotide and amino acid sequences of all constructs used in the present study, omitting both the *pro*-peptide sequence and the native signal peptide, are available in Additional File 1 (Figures S1-S12).

All cloning steps were performed using standard molecular biology techniques. A list of the oligonucleotide primers used is given in Table S1 (Additional File 1). Correct insertion of the GalOx-encoding genes and the absence of mutations were checked by DNA sequencing using the primers T7fwd and T7rev for the *E. coli *constructs or 5'AOX1 and 3'AOX1 for the *P. pastoris *constructs, respectively (Table S1, Additional File 1). Nucleotides, buffers and enzymes were purchased from Fermentas (Stockholm, Sweden).

#### Expression in *E. coli*

The wild-type *galox *gene from *Fusarium graminearum *(wt*galox*) and a variant optimized for the expression in *E. coli *(M1*galox*) were generously provided by Prof. Nicholas Turner (University of Manchester, UK). The M1 variant differs from the wild-type in 5 amino acid substitutions (S10P, M70V, G195E, V494A, N535D), which have shown beneficial effects on the expression level and the stability of GalOx in *E. coli *[17,18]. The two genes, coding only for the mature GalOx protein, were cloned into a pET16b^+ ^vector (Novagen, Darmstadt, Germany) with an N-terminal His_10_-tag and an IPTG-inducible T7 promoter for the intracellular expression in *E. coli *[18].

#### Expression in *P. pastoris*

To test the intracellular expression in *P. pastoris*, both *galox *genes were cloned into the pPIC3.5 vector (Invitrogen, CA, USA) using the restriction sites *Avr*II and *Not*I. For extracellular expression the genes were cloned into the pPICZα-C vector (Invitrogen), providing the α-factor signal sequence for secretion, using the restriction sites *Not*I and *Xba*I. For all these constructs, integration of the *galox *gene occurred at the AOX1 locus in the genome of *P. pastoris*. To check the effect of a His-tag on the expression level and the activity of GalOx, different constructs were prepared either with or without an N-terminal His_10_-tag following the α-factor secretion signal peptide. A *galox *gene codon-optimized for the expression in the yeast *P. pastoris *(Additional File 1, Figure S13), was synthesized (GenScript, NJ, USA) and cloned into the pPICZα-C vector with or without a C-terminal His_6_-tag using the restriction sites *Not*I and *Xba*I. The optimization parameters were determined according to the OptimumGene™ algorithm (GenScript, NJ, USA) taking into account the e.g. codon usage bias, GC content, mRNA secondary structures and RNA instability motifs.

### Media and strains

*E. coli *TOP10 cells were used for maintenance and propagation of all plasmids. For expression trials in bacteria, the *E.coli *strain BL21DE3 was used. Intracellular expression in *P. pastoris *was performed in the strain GS115 and for extracellular expression the protease-deficient *P. pastoris *strain SMD1168H was used. Names of all recombinant strains are given in Table 1. A 3-letter code following the standard organism binomial nomenclature describes the features of the constructs: F, wild-type *galox *gene from *F. graminearum*; E, *galox *gene optimized for the expression in *E. coli*; P, *galox *gene optimized for the expression in *P. pastoris*; N, N-terminal His_10_-tag; x, no His-tag; C, C-terminal His_6_-tag; I, intracellular expression; E, extracellular expression.

**Table 1:** Expression hosts, *galox *genes and vectors used in this study

Strain name	Host organism	Vector	*galox *Gene construct	Polyhistidine tag	Localization
*E. coli *FNI	*E. coli *BL21DE3^a^	pET16b^+^	*Fusarium *wild-type	N-terminal His_10_	intracellular
*E. coli *ENI			*E. coli*-optimized	N-terminal His_10_	

*P. pastoris *FxI	*P. pastoris *GS115^b^	pPIC3.5	*Fusarium *wild-type	None	intracellular
*P. pastoris *ExI			*E. coli*-optimized	None	
*P. pastoris *FNI			*Fusarium *wild-type	N-terminal His_10_	
*P. pastoris *ENI			*E. coli*-optimized	N-terminal His_10_	

*P. pastoris *FxE	*P. pastoris *SMD1168H^c^	pPICZα-C	*Fusarium *wild-type	None	extracellular
*P. pastoris *ExE			*E. coli*-optimized	None	
*P. pastoris *FNE			*Fusarium *wild-type	N-terminal His_10_	
*P. pastoris *ENE			*E. coli*-optimized	N-terminal His_10_	
*P. pastoris *PxE			*Pichia*-optimized	None	
*P. pastoris *PCE			*Pichia*-optimized	C-terminal His_6_	

a. Genotype: F^- ^ompT gal dcm lon hsdS_B_(r_B_^- ^m_B_^-^) λ(DE3 [lacI lacUV5-T7 gene 1 ind1 sam7 nin5]) [33]

b. Genotype described in ref. [34,35]

c. Genotype described in ref. [35]

For propagation of the different constructs, *E. coli *cells were cultivated in LB-medium (yeast extract 5 g·L^-1^; tryptone 10 g·L^-1^; NaCl 10 g·L^-1^; pH 7.5) or Low Salt LB-medium (yeast extract 5 g·L^-1^; tryptone 10 g·L^-1^; NaCl 5 g·L^-1^; pH 7.5) under appropriate selective conditions (ampicillin or zeocin was added to a final concentration of 0.1 g·L^-1 ^or 0.025 g·L^-1^, respectively) according to the manufacturer's protocol (Invitrogen). Expression studies in *E. coli *were performed in TB_amp_-medium (yeast extract 24 g·L^-1^; peptone from casein 12 g·L^-1^; glycerol 4 mL·L^-1^; KH_2_PO_4_-buffer 1 M, pH 7.5; ampicillin 0.1 g·L^-1^).

*P. pastoris *clones were propagated in YPD medium (yeast extract 10 g·L^-1^; peptone 20 g·L^-1^; dextrose 10 g·L^-1^) under appropriate selective conditions. After transformation of the *P. pastoris *strain GS115 with linearized pPIC3.5/*galox *constructs, cells were spread on RDB_amp _(Regeneration Dextrose Base) plates and incubated at 30°C for 48 h. SMD1168H cells were transformed with linearized pPICZα-C/*galox *constructs and grown on YPDS_zeocin _plates at 30°C for 48 h. Expression experiments were performed using Buffered Glycerol-Complex Medium (BMGY) and Buffered Methanol-Complex Medium (BMMY). All media were prepared according to the manufacturer's protocol (Invitrogen).

### Screening for His^+^Mut^+ ^or His^+^Mut^S ^variants in GS115

Correctly sequenced pPIC3.5/*galox *constructs were linearized with *Sac*I and transformed into the *P. pastoris *strain GS115, resulting in His^+^Mut^+ ^transformants. To guarantee the correct insertion in the genomic DNA, 10 colonies of each transformation were tested for phenotype and growth on Minimal Methanol (MM) and Minimal Dextrose (MD) plates according to the manufacturer's protocol (Invitrogen).

### Colony screening for highly-expressing *P. pastoris *transformants

To test the expression level in *P. pastoris *and to find a highly-expressing clone, 5 colonies of each transformation were picked and small-scale expression trials were performed. Typically (in ca. 10 independent transformations), best levels of activity were observed in two of five clones, and very high producing outliers were not observed. Transformants were precultured in 25 mL BMGY at 30°C and 220 rpm overnight. The following day, an appropriate amount of preculture was sampled, cells were spun down and diluted in 10 mL BMMY to a final OD_600 _of 1.0. Cultures were grown in 50 mL Falcon tubes at 25°C and 220 rpm for 120 h and methanol (MeOH) was reconstituted to a final concentration of 0.5% every day. Samples were taken periodically and analyzed for protein content and enzymatic activity and aliquots were loaded onto SDS-PAGE gels.

### Shake-flask cultivation of GalOx in *E.coli *BL21DE3

Cultures (1 liter) of *E. coli *BL21DE3 transformants were grown in TB_amp _medium in baffled flasks at 37°C and 220 rpm. Protein expression was induced at an OD_600 _of ~0.5 by adding IPTG to a final concentration of 0.5 mM. After incubation at 25°C for further 20 h, approximately 15 g of wet biomass per liter were harvested by centrifugation at 10,000×g for 15 min and 4°C, and resuspended in Buffer A (NaH_2_PO_4 _50 mM; NaCl 500 mM; imidazole 20 mM; pH 7.5) containing the protease inhibitor PMSF (0.1% w/v). After disruption in a French Press (1200 psi) the crude cell extract was separated from cell debris by centrifugation (70,400×g, 4°C) and used for protein purification by immobilized metal affinity chromatography (IMAC) with a 10 mL Ni-charged Sepharose 6 Fast Flow Resin (GE Healthcare; Uppsala, Sweden). Before the sample was loaded, the column was equilibrated with 10 column volumes (CV) of buffer A. After the protein sample was applied to the column, it was washed with 3 CV of the same buffer, before proteins were eluted with a linear gradient (0-1 M imidazole) of 5 CV Buffer B (NaH_2_PO_4 _50 mM; NaCl 500 mM; imidazole 1 M; pH 7.5). Active fractions were combined and imidazole was removed by ultrafiltration using an Amicon Ultra Centrifugal Filter Device (Millipore; Billerica, MA, USA) with a 10-kDa cut-off membrane. The concentrated enzymes were washed 3 times with 10 mL of NaH_2_PO_4_-buffer (100 mM, pH 7.5) and finally diluted in the same buffer to a total protein concentration of 2-3 mg·mL^-1^.

### Intracellular and extracellular large scale expression of GalOx in *P. pastoris*

Highly-expressing *P. pastoris *transformants were precultured in 250 mL BMGY in 2 L baffled flasks at 30°C overnight. The next day, OD_600 _was measured, an appropriate amount of culture was sampled, cells were spun down and diluted in 1 liter BMMY to an initial OD_600 _of 1.0. Cultures were grown in special 2.5 L baffled flasks (Tunair; Sigma-Aldrich; Stockholm, Sweden) at 25°C and 220 rpm up to 216 h. To optimize the expression of active GalOx, different constructs with either a N-terminal, a C-terminal or no His-tag and different temperatures (25°C and 30°C) during cultivation were tested. In addition, the expression of correctly folded enzyme was tested in the presence of copper (II) ions (as CuSO_4_), which is required for the activation of GalOx [5,19,21], at a concentration of 0.5 or 1.0 mM in the medium.

To obtain the intracellularly expressed GalOx, *P. pastoris *cells were were harvested by centrifugation (4,000×g, 4°C, 15 min) and resuspended in sodium phosphate buffer (100 mM, pH 7.5) containing the complete-mini EDTA-free protease inhibitor (1 tablet per 10 mL buffer; Roche; Basel, CH). *P. pastoris *cells were disrupted in a French Press (1200 psi) and cell debris was separated by subsequent centrifugation (70,400×g, 4°C, 30 min).

### Analysis of expression- and growth-parameters

Samples were taken in appropriate time intervals during cultivations and analyzed for optical density at 600 nm (OD_600_), dry cell weight (DCW) and concentration of expressed protein and enzymatic activity. To determine the DCW, 1 mL samples were taken, biomass was harvested in a previously dried and pre-weighed tube by centrifugation (4000 rpm, 5 min), supernatants were discarded, cells were washed twice with ultrapure H_2_O and then dried at 65°C to a constant weight. To evaluate the process conditions, the volumetric productivity (enzyme activity units per liter culture per hour of cultivation, U· L^-1^·h^-1^) and the product yield (expressed as the mass of protein produced per mass of DCW, g·g^-1^) were determined.

### Enzyme activity assay

GalOx activity was measured with the standard chromogenic ABTS [2,2'-azinobis(3-ethylbenzthiazolinesulfonic acid)] assay [20]. A sample of diluted enzyme (10 μL) was added to 990 μL of assay buffer containing horseradish peroxidase (142 U), ABTS (14.7 mg), NaH_2_PO_4_-buffer (100 mM, pH 7.5) and D-galactose (300 mM). The absorbance change at 420 nm (ε_420 _= 42.3 mM^-1 ^cm^-1^) was recorded at 30°C for 180 s. One Unit of GalOx activity was defined as the amount of enzyme necessary for the oxidation of 2 μmol of ABTS per min, corresponding to the consumption of 1 μmol of O_2 _per min. Protein concentrations were determined at 595 nm by the Bradford assay [22] using the BioRad Protein Assay Kit with BSA as standard.

### Posttranslational processing of GalOx

The native GalOx enzyme is expressed as a precursor peptide with an additional 17-amino acid pro-sequence, lacking the thioether bond between Tyr272 and Cys228. In this study, GalOx was heterologously expressed in different hosts without the pro-sequence at the N-terminus. To find the optimal conditions for post-translational activation, different amounts of CuSO_4 _were added either to the concentrated enzyme preparations or directly into the cultivation media. After incubation, excess copper was removed by gel filtration using PD10 desalting columns (GE Healthcare), and enzymes were tested for catalytic activity and protein content and analyzed on SDS-PAGE gels.

### Activation studies

To test if the stored enzyme preparations could be further activated by the additional incubation with an oxidizing agent [5,14,17,23,24], various experiments with K_3_Fe(CN)_6 _and CuSO_4 _were performed. Aliquots of GalOx were incubated with either 5 mM, 50 mM and 100 mM K_3_Fe(CN)_6 _or with additional 0.5 mM CuSO_4 _on ice for either 10 min, 1 hour or overnight. After removal of the excessive oxidizing agent by gel filtration using PD10 desalting columns (GE Healthcare), catalytic activity with D-galactose (300 mM) and the protein content were measured.

### Electrophoresis

To check the purity of the enzyme preparations and the correct formation of the radical Tyr-Cys cofactor [5], SDS-PAGE analysis was done. The heterologously expressed GalOx in this study can run as 2 distinct bands on an SDS-PAGE gel: at 65.5 kDa (mature GalOx containing the thioether bond) and at 68.6 kDa (mature GalOx lacking the thioether bond). SDS-PAGE was performed using precast 10% Bis-Tris gels (Invitrogen) and 1x MOPS-buffer (MOPS 10.46 g·L^-1^; EDTA 0.3 g·L^-1^; TrisBase 6.06 g·L^-1^; SDS 1.0 g·L^-1^; pH 7.7). Gels were run in the Novex MiniCell (Invitrogen) at 150 V for about 2 h. The protein mass standard used was the SeeBlue Plus 2 prestained standard (Invitrogen). Gels were stained with Coomassie blue.

### Western blotting

To perform Western blotting analysis, an SDS-PAGE gel was run with 10 μL-aliquots of a GalOx expressed with a C-terminal His_6_-tag (2 mg·mL^-1^; pure and 1:10 dilution). Proteins were transferred to a nitrocellulose membrane (Scheichler & Schuell Protran BA83 0.2 μM) using Towbin buffer [25] and a Trans-Blot apparatus (Biorad; Sundbyberg, Sweden). The transfer was carried out at 40 V overnight under gentle stirring at 4°C. The membrane was blocked in 1xTris-buffered saline with Tween 20 (TBST; 100 mM Tris-HCl, 150 mM NaCl, pH 7.5, 0,1% Tween 20) supplemented with 5% BSA at room temperature for 2 hours and then incubated with a 1:5000 dilution (v/v) of an anti His_6 _antibody conjugated to horse radish peroxidase (Pierce, Biocompare; CA, USA) at room temperature with gentle rocking for another 2 hours. The membrane was extensively washed in TBST and then revealed using the ECL kit (Amersham; Uppsala, Sweden). The images were acquired with a GelDoc camera (Biorad) using the chemiluminescence filter.

### Storage stability of GalOx preparations from *P. pastoris*

To find the best conditions to store the large amounts of GalOx produced in *P. pastoris *for an extended period of time, different conditions were tested. Enzyme solution was aliquoted in 500 L portions and either stored at 4°C, snap-frozen in liquid N_2 _and stored at -80°C or lyophilized and stored at -20°C. After different time intervals samples were thawed on ice and catalytic activity and protein content were measured. Storage at 4°C was tested for 4 weeks, and samples were analyzed every day. Lyophilized samples were resuspended in the same amount of buffer (500 μL) and the activity at a substrate concentration of 300 mM D-galactose were determined and the protein content were compared to the values before lyophilisation.

## Results and Discussion

In this study, different strategies for the recombinant production of GalOx were compared to systematically explore the advantages and potential drawbacks of each system. An overview of the strain names, the GalOx gene constructs, expression vectors, and host organisms is given in Table 1.

*F. graminearum *GalOx is naturally produced as a *pre-pro*-enzyme containing a 17 amino acid N-terminal sequence that is cleaved to yield the "immature" form of the enzyme. A previous study suggested that the *pro*-sequence was required as an intramolecular chaperone for copper binding and cofactor formation [24]. In contrast, however, fully active GalOx has been successfully produced in a number of cases by aerobic addition of copper (II) to versions of GalOx lacking this pro-sequence [5,19,21]. Additionally, *Pichia pastoris *is not able to efficiently process the native signal sequence [26]. Thus, all gene constructs in this study were designed to omit both the *pro*-peptide sequence and the native signal peptide (see Additional File 1, Figure S1-S12).

### Intracellular expression of GalOx in *E. coli*

To quantify the expression of active GalOx from *F. graminearum *in the prokaryotic host *E. coli*, 2 genes, the wild-type *galox *gene (wt*galox*) and the optimized variant M1 (M1*galox*), were cloned into a pET16b^+ ^vector and transformed into the *E. coli *strain BL21DE3 to give *E. coli *FNI and *E. coli *ENI, respectively (Table 1). The optimized gene M1 differs in 5 amino acid substitutions (see Additional File 1), which have been reported to result in a higher expression yield and increased stability of the fungal enzyme in *E. coli *[17,18]. In these studies, a higher expression of GalOx in *E. coli *was desired for high-throughput screening in the context of directed evolution studies for altering the substrate specificity of this enzyme [17,18]. However, a detailed analysis and explicit comparison of the expression levels of these 2 genes in *E. coli *has not been performed previously.

Both the wild-type and M1-variant genes used in this study encoded the mature GalOx protein, in which the native signal sequence and the pro-sequence were replaced with an N-terminal His_10_-tag. Following successful expression of both constructs in *E. coli *and cell disruption in a French Press, GalOx was purified from the crude extracts using IMAC. The resulting purification factor was 93.8 for the wild-type enzyme and 109.0 for the optimized variant M1. The expression level of the optimized gene was 10-fold higher than that of wt*galox *(4.1 mg·mL^-1^and 0.4 mg·mL^-1^were obtained, respectively). Likewise, the specific activity of the purified M1 product was higher by a factor of 2.4 (24 U·mg^-1 ^versus 10 U·mg^-1 ^for wt*galox*), prior to further activation with copper (II) ions.

**Figure 2:**
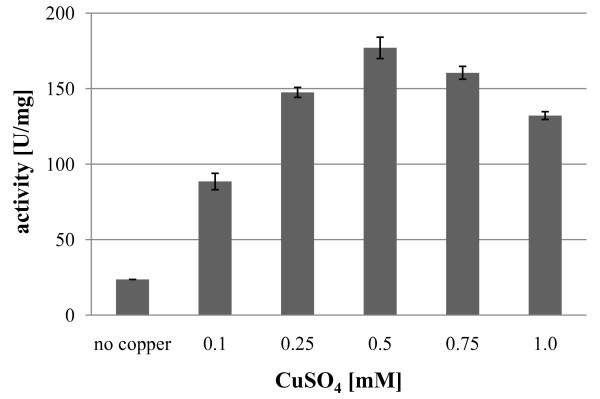
**Analysis of the optimal concentration for the post-translational activation with CuSO**_**4 **_**of GalOx expressed in *E. coli***.

**Figure 3:**
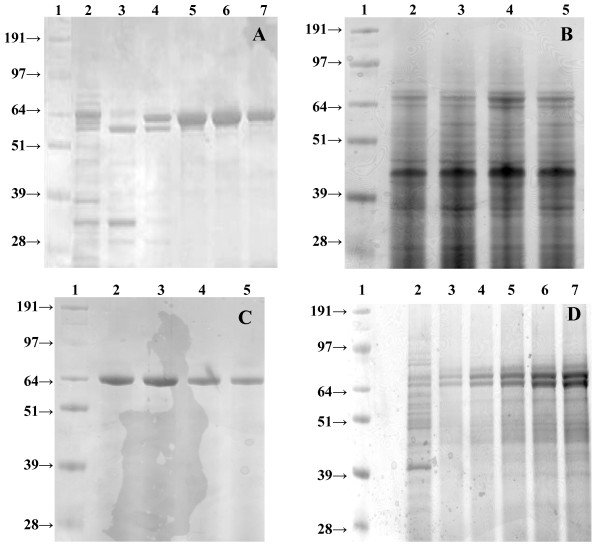
**SDS-PAGE analyses of recombinantly produced GalOx **. A. Fractions collected from the IMAC purification of GalOxM1 expressed intracellularly in *E. coli *at 25°C (incubated with 0.5 mM CuSO_4 _before loading on the gel); lane 1, molecular mass standard; lane 2, crude extract; lane 3, flow-through; lane 4-7, different fractions of GalOxM1 eluted with a linear gradient of imidazole (20 mM to 1 M imidazole within 5 column volumes). B. Crude extracts obtained after 120 h cultivation of *P. pastoris *expressing the wt*galox *or the variant M1*galox *either with or without an N-terminal His_10_-tag at 25°C (intracellular production); lane 1, molecular mass standard; lane 2, crude extract of His_10_-wtGalOx; lane 3, crude extract of His_10_-M1GalOx; lane 4, crude extract of wtGalOx; lane 5, crude extract of M1GalOx. C. Aliquots of the supernatant obtained at different time points during the cultivation of *P. pastoris *expressing the wt*galox *gene from *F. graminearum *without the N-terminal His_10_-tag extracellularly at 25°C; lane 1, molecular mass standard; lane 2-5, 48 h; 72 h; 96 h (1:10 dilution); 120 h (1:10 dilution). D. Aliquots of the supernatant obtained at different time points during the cultivation of *P. pastoris *expressing the wt*galox *gene from *F. graminearum *without the N-terminal His_10_-tag extracellularly at 30°C; lane 1, molecular mass standard; lane 2-7, 12 h; 24 h; 48 h; 72 h; 96 h; 120 h.

To analyze the optimal conditions for the post-translational activation of the immature GalOx with copper (II) ions [5,19,21], the purified enzyme variant GalOxM1 was incubated with different concentrations of CuSO_4 _at 4°C and stirring overnight. After incubation, excess copper was removed by gel filtration and enzymatic activity was measured. Incubation with 0.5 mM CuSO_4 _resulted in the highest activation of GalOx (Figure 2), the specific activity of purified GalOxM1 increased 7.5-fold from 24 U·mg^-1 ^to 180 U·mg^-1^. This latter specific activity value is equivalent to a *v*_o _[E]_t_^-1 ^value of 190 s^-1^, which was obtained at 20× *K*_m _(300 mM Gal, see Materials and Methods) [18]. This value compares favorably with the *k*_cat _value of 156 s^-1 ^previously determined for this enzyme variant [18]. Thus, the kinetic data indicate that the degree of enzyme activation may supercede that obtained in previous studies. To check the purity of the enzyme and the correct formation of the radical cofactor in the active site [5,24], aliquots of the purified enzyme were loaded onto an SDS-PAGE gel (Figure 3A). A band at an apparent mobility of 65 kDa, which corresponds to the mature GalOx containing the correctly formed Tyr-Cys radical, can be seen in lanes 4-7. It has been observed previously that activation of the immature form of GalOx (68 kDa) causes a mobility shift in SDS-PAGE, which can be used as an indicator of the degree of activation [5,24].

Clearly, GalOx proteins lacking the pro-sequence can be successfully processed post-purification by the addition of 0.5 mM CuSO_4 _under aerobic conditions to yield the catalytically active, mature species. Using this strategy, after 20 h of expression at 25°C, 3600 U·L^-1 ^active GalOxM1 could be obtained, resulting in a volumetric productivity of 180 U·L^-1^·h^-1 ^for this expression system under these conditions. This expression level of the fungal enzyme GalOx in the prokaryotic host *E. coli *thus forms a platform for directed evolution studies of this enzyme [17,18], where a rapid, easily implemented expression system is advantageous.

### Intracellular expression of GalOx in *P. pastoris*

The methylotrophic yeast *Pichia pastoris *has become one of the leading eukaryotic expression systems for general laboratory use due to its ease of cultivation and the availability of a range of commercially available expression vectors [27,28]. Indeed, strain and vector systems have been developed, which allow both intra- and extracellular protein production under the control of various promoters, the most common being the methanol-induced *alcohol oxidase 1 *(*AOX1*) promoter [29].

To examine the intracellular expression in the eukaryotic host *P. pastoris*, the wt*galox *gene and the variant M1*galox *were cloned into the pPIC3.5 vector, with or without an N-terminal His_10_-tag, and transformed into the *P. pastoris *strain GS115 to give *P. pastoris *strains FNI, ENI, FxI and ExI (Table 1). Clones selected for highest expression level of GalOx were subsequently cultivated at 1 L-scale at 25°C over a period of five days to monitor enzyme production (Table 2 and Figure 3B).

**Table 2:** Volumetric activity of GalOx expressed intracellularly in *P. pastoris*

	**vol. activity**^b ^**[U·mL**^**-1**^**]**					
**strain**^**a**^	**24 h**	**48 h**	**72 h**	**96 h**	**120 h**	**120 h**^*c*^

*P. pastoris *FxI	3.2	7.4	11.0	14.0	14.0	0.9
*P. pastoris *ExI	1.7	4.2	7.1	10.0	11.0	0.8
*P. pastoris *FNI	0.7	1.3	1.9	2.4	2.5	0.5
*P. pastoris *ENI	0.6	1.0	1.8	2.2	2.2	0.4

^a ^See Table 1

^b ^vol.activity of GalOx after incubation with 0.5 mM CuSO_4 _at 4°C and stirring overnight; wild-type GalOx from *F. graminearum *and the variant M1 with or without an N-terminal His_10_-tag were expressed intracellularly in *P. pastoris *GS115 at 25°C for 120 hours

^c ^vol. activity [U·mL^-1^] before the incubation with 0.5 mM CuSO_4_

The expression of the wt*galox *gene from the fungus *F. graminearum *in the yeast *P. pastoris *was slightly better than the M1*galox *gene, which had been optimized for the expression in *E. coli *(Table 2). The incubation with 0.5 mM CuSO_4 _efficiently activated the GalOx enzyme in the crude extracts and resulted in an increase of activity up to 15-fold, with lower degrees of activation observed in variants bearing a N-terminal His_10_-tag. The wt*galox *gene yielded the highest amount of expressed, active GalOx enzyme and a volumetric productivity of 120 U·L^-1^·h^-1^. Notably, the presence of an N-terminal His_10_-tag on both the wild-type and M1 variants severely suppressed the yield of enzyme obtained in cultivations (Table 2).

As shown in Figure 3B, bands at 65 kDa, corresponding to the mature GalOx enzyme containing the Tyr-Cys cofactor, but also bands at 68 kDa, possibly corresponding to the pre-mature GalOx lacking the radical cofactor in the active site [5,24], could be detected. However, incubation with up to 2.0 mM CuSO_4 _did not result in an increased amount of active GalOx, but rather in a decrease in protein content after the incubation because of protein denaturation. As a consequence of the intracellular production, many other proteins were detected in the crude extracts (Figure 3B), which could also influence the copper-activation of GalOx and further complicate subsequent purification in the absence of an affinity tag. Generally speaking, the lower volumetric productivity of the intracellular *P. pastoris *system indicates that this system is not competitive with intracellular expression in *E. coli*.

### Extracellular expression of GalOx in *P. pastoris*

One of the key advantages of *P. pastoris *for recombinant protein production lies in the ability of this yeast to secrete proteins to the culture medium under the agency of the *Saccharomyces cerevisiae *α-factor secretion signal peptide [27,30]. *P. pastoris *in general secretes low levels of endogenous proteins, and the SMD1168H strain in particular has low detrimental protease activity, thus facilitating the recovery and downstream processing of reasonably pure crude extracts. For comparison with the *E. coli *and *P. pastoris *intracellular production systems, the expression of the wild-type, M1 (*E. coli *optimized) and *P. pastoris *codon-optimized variant of *galox *was examined in combination with various His affinity tag strategies (Table 1).

#### Effect of N-terminal His_10_-tag on expression of wtgalox and M1galox

The wt*galox *gene and the optimized variant M1*galox *were cloned into the pPICZα-C vector with or without an N-terminal His_10_-tag and transformed into the *P. pastoris *strain SMD1168H to give *P. pastoris *strains FNE, ENE, FxE and ExE (Table 1). After colony screening, clones with the highest expression level of GalOx were cultivated in shake-flask cultivations (1 L) at 25°C for 120 h and analyzed periodically. After determining the optimal amount of CuSO_4 _for activation (data not shown, similar to Figure 2), enzymatic activity was measured in the crude extracts before and after incubation with 0.5 mM CuSO_4 _and stirring overnight.

After 120 h of cultivation, incubation with CuSO_4 _increased GalOx activity up to 14-fold (Table 3). As observed before for the intracellular expression in *P. pastoris*, the presence of the N-terminal His_10_-tag in both gene variants significantly reduced enzyme production; the wt*galox *gene from *F. graminearum *without the N-terminal His_10_-tag resulted in the highest amount of active GalOx enzyme and a volumetric productivity of 610 ± 30 U·L^-1^·h^-1 ^(Table 3). As in the intracellular cases, the wt*galox *gene from *F. graminearum *yielded more enzyme in *P. pastoris *than the M1*galox *gene, which had been optimized for *E. coli *expression. The resulting preparations had a specific activity of 260 ± 14 U mg^-1 ^(Table 4). This equates to a *v*_o _[E]_t_^-1 ^value of 280 s^-1^, which was obtained at 300 mM Gal (3.6× *K*_m _[4], see Materials and Methods). Assuming standard Michaelis-Menten kinetics, at this substrate concentration the *v*_o _[E]_t_^-1 ^value is ca. 78% of the *k*_cat _value. The *k*_cat _value for recombinant, wild-type GalOx from *F. graminearum *has been previously determined to be 503 ± 16 s^-1 ^[4], which implies that GalOx produced by strain *P. pastoris *FxE (Table 1) is ca. 70% active. SDS-PAGE analysis (Coomassie staining, Figure 3C) of the cultivation broth at different time points indicated, however, that a single protein band at 65 kDa was produced, corresponding to the mature GalOx with the correctly formed Tyr-Cys cofactor [5,24]. As GalOx constituted for the majority of the secreted proteins in these samples, a simple purification by (NH_4_)_2_SO_4 _precipitation, dialysis, and ultrafiltration was performed. However, media components in the concentrated enzyme preparation led to a brown color, which hindered the analysis of the oxidation state of the enzyme by visible spectroscopy (445 nm, [5,17]).

**Table 3:** Catalytic activity and protein content of GalOx expressed extracellularly in *P. pastoris*

	**vol. activity**^b ^**[U·mL**^**-1**^**]**							
**strain**^**a**^	**24 h**	**48 h**	**72 h**	**96 h**	**120 h**			**120 h**^c^

					vol. activity [U·mL^-1^]	protein [mg·mL^-1^]	spec. activity [U·mg^-1^]	
						
*P. pastoris *FxE	15.2	29.0	55.0	69.0	73.0	0.28	260	6.1
*P. pastoris *ExE	15.0	27.0	43.0	60.0	61.0	0.24	254	4.3
*P. pastoris *FNE	5.3	6.0	6.2	7.6	7.3	0.12	61	1.1
*P. pastoris *ENE	3.0	5.6	5.7	5.9	6.1	0.10	61	1.0

^a ^See Table 1

^b ^catalytic activity and protein content after incubation with 0.5 mM CuSO_4 _at 4°C and stirring overnight; wild-type GalOx from *F. graminearum *and the variant M1 with or without an N-terminal His_10_-tag were expressed extracellularly in *P. pastoris *SMD1168H at 25°C for 120 hours

^c ^vol. activity [U·mL^-1^] before the incubation with 0.5 mM CuSO_4_

**Table 4:** Summary of productivity of galactose oxidase in different hosts

**strain**^**a**^	specific activity **[U·mg**^**-1**^**]**	volumetric productivity **[U·L**^**-1**^**·h**^**-1**^**]**	specific productivity **[mg product·g**^**-1 **^**biomass·h**^**-1]**^
*E.coli *ENI	180 ± 7 (n = 2)^b^	180 ± 7	0.15 ± 0.1
*P. pastoris *FxI^c^	10 (n = 1)	120	n.d.^d^
*P. pastoris *FxE^c^	260 ± 14 (n = 3)	610 ± 30	0.12 ± 0.09
*P. pastoris *PCE	250 ± 12 (n = 3)	580 ± 25	0.19 ± 0.1

^a ^See Table 1

^b ^n, number of replicates

^c ^GalOx was not purified from crude extract

^d ^n.d. not determined

#### Effect of cultivation temperature on enzyme yield

**Figure 4:**
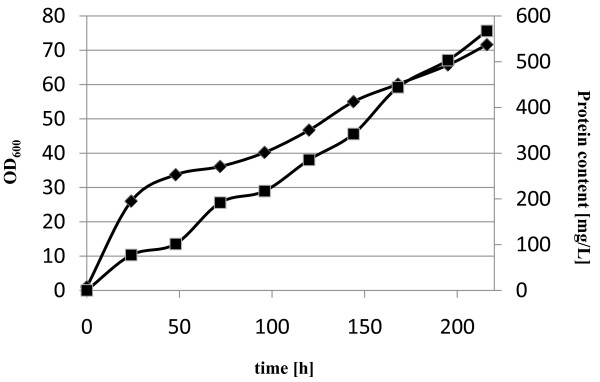
Time course of biomass and protein content during cultivation of *P. pastoris *expressing the wtGalOx from *F. graminearum *extracellularly at 25°C for 216 h. ■, protein content [mg·L^-1^]; ♦, OD_600_

Since the volumetric productivity was more than 3-fold higher for the extracellular expression of the wt*galox *gene (without His tag) in *P. pastoris *compared to the production of GalOx in *E. coli*, we chose this system to test different cultivation conditions. After 120 h of cultivation at 25°C, the OD_600_, the DCW and the total protein content in the medium were still increasing, implying that the culture had still not reached the stationary phase yet (graphs not shown). Therefore we performed 2 cultivations in parallel, at 25°C and the more commonly used 30°C [31], for an extended time of 216 h. After 216 h of cultivation and activation of the enzyme with 0.5 mM CuSO_4 _overnight, 150 U·mL^-1 ^GalOx were obtained at 25°C, whereas only 56 U·mL^-1 ^were obtained at 30°C. The protein contents in the media were comparable for both temperatures (0.57 mg·mL^-1 ^at 25°C and 0.52 mg·mL^-1 ^at 30°C, respectively) resulting in specific activities of 254 U·mg^-1 ^at 25°C and 108 U·mg^-1 ^at 30°C, respectively. However, as shown in Figure 3D, *P. pastoris *secreted a more complex mixture of proteins into the medium (*cf. *Figure 3C). The presence of strong bands with apparent mobilities of 65 kDa and 68 kDa suggests that the higher temperature resulted in partly misfolded constructs, which could not be correctly activated to generate the Tyr-Cys radical co-factor by the addition of copper (II) ions under aerobic conditions. A similar negative effect of increasing the cultivation temperature on production yield of *P. pastoris *has been observed previously [26], thus suggesting that lower-temperature production strategies are to be preferred. Interestingly, even after 216 h of cultivation at 25°C, the OD_600 _value and the protein content in the medium were still increasing (Figure 4). However, for practical reasons, cultivation times of more than 216 h (9 d) were not tested.

#### Direct activation of GalOx in the cultivation medium

To streamline the extracellular production of GalOx by *P. pastoris*, the direct activation of GalOx by supplementing copper (II) in the culture medium was tested. Transformants were cultivated in BMMY supplemented with CuSO_4 _to final concentrations of 0.5 and 1.0 mM (2 mM Cu^++ ^is known to result in growth toxicity to *P. pastoris *[32]). *P. pastoris *resulted in a final OD_600 _between 65-70 and a final dry cell weight between 45-50 mg·mL^-1 ^after 216 hours regardless of the presence of copper in the medium. The protein content in the medium was also comparable between the different cultivations; without copper in the medium a protein concentration of 0.57 mg·mL^-1 ^was obtained, and in the presence of 0.5 and 1.0 mM copper 0.54 mg·mL^-1 ^and 0.50 mg·mL^-1 ^could be measured, respectively.

**Figure 5:**
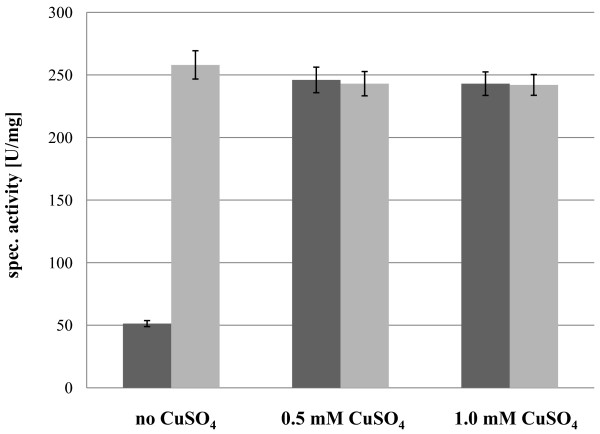
**Specific activity of wtGalOx from *F. graminearum *expressed extracellularly in *P. pastoris *at 25°C for 216 h **. The cultivation medium BMMY was either not supplemented with additional CuSO_4 _or cells were cultivated in the presence of either 0.5 mM or 1.0 mM CuSO_4_. After 216 h of cultivation the specific activity of GalOx in the supernatants was determined (dark grey bars). Additional 0.5 mM CuSO_4 _were added and the samples were incubated at 4°C and stirring overnight. After removal of excessive CuSO_4 _by gel filtration, the specific activity was determined again (light grey bars). Error bars represent standard deviation for 3 biological replicates and 2 assay replicates each.

As desired, addition of copper (II) in the growth medium resulted in the immediate activation of GalOx (Figure 5). Subsequent incubation of the enzyme with additional 0.5 mM CuSO_4 _at 4°C and stirring overnight did not result in further activation, implying that the presence of copper in the cultivation medium was sufficient for effective processing of GalOx. Assuming 100% activation in all cases, the protein concentration values indicate a slightly reduced productivity with increasing concentrations of copper (II) ions in the medium, perhaps due to metal ion toxicity. The highest amount of active GalOx, and therefore the highest volumetric productivity of 610 U·L^-1^·h^-1^, was obtained when *P. pastoris *was cultivated without additional CuSO_4 _in the medium and GalOx was subsequently activated by 0.5 mM CuSO_4_. Consequently, post-harvest activation of GalOx with copper (II) was used for further experiments.

#### Codon-optimization of GalOx for P. pastoris and effect of C-terminal His_6_-tag addition

To test the possibility of further improving the extracellular expression of GalOx in *P. pastoris*, a codon-optimized variant for *P. pastoris *was obtained by gene synthesis. The sequence identity between the wt*galox *gene from *F. graminearum *and the codon-optimized gene for *P. pastoris *was 75% (Figure S13; Additional File 1). Due to the observation that the N-terminal His_10_-tag negatively affected the production of GalOx in *P. pastoris *(both intra- and extracellular), the codon-optimized *galox *gene was cloned into the pPICZα-C system with and without a C-terminal His_6_-tag. Both constructs were transformed into the *P. pastoris *strain SMD1168H to give *P. pastoris *PCE and *P. pastoris *PxE, respectively (Table 1). After screening for highly-producing clones, shake-flask cultivations of *P. pastoris *FxE, *P. pastoris *PxE, and *P. pastoris *PCE were performed in parallel at 25°C for 120 h.

**Figure 6:**
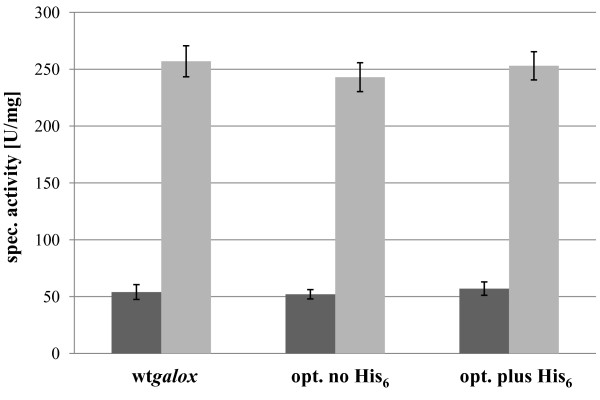
**Specific activitiy of wtGalOx from *F. graminearum *or a codon-optimized variant with or without a C-terminal His**_**6**_**-tag expressed extracellularly in *P. pastoris *at 25°C for 120 h **. After 120 h of cultivation the specific activity of GalOx in the supernatants was determined (dark grey bars). Additional 0.5 mM CuSO_4 _were added and the samples were incubated at 4°C and stirring overnight. After removal of excessive CuSO_4 _by gel filtration, the specific activity was determined again (light grey bars). Error bars represent standard deviation for 2 assay replicates.

**Figure 7:**
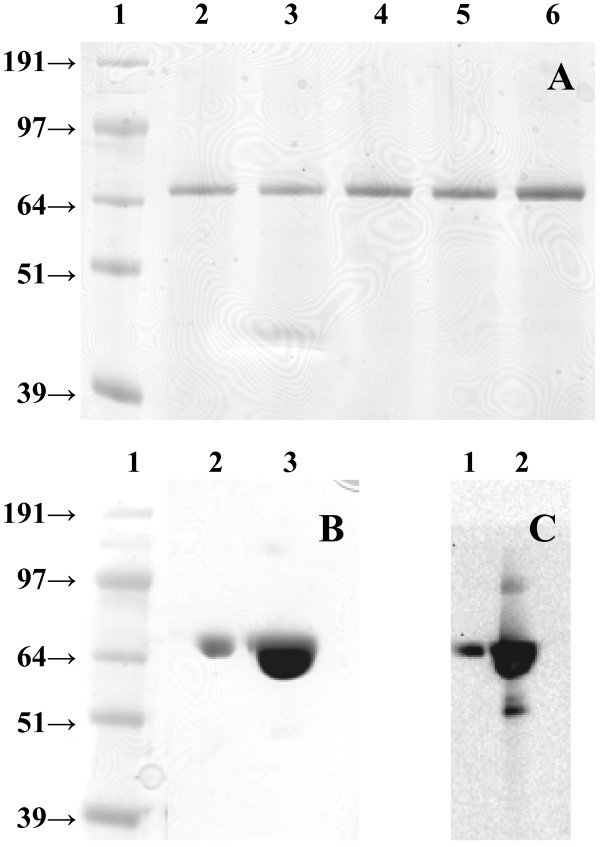
**SDS-PAGE analysis and Western blot of the *Pichia*-codon-optimized GalOx gene with a C-terminal His**_**6**_**-tag expressed extracellularly in *P. pastoris *at 25°C **. A. SDS-PAGE analysis of aliquots of the supernatant obtained at different time points during the cultivation of *P. pastoris *expressing the codon-optimized GalOx gene with a C-terminal His_6_-tag extracellularly at 25°C; lane 1, molecular mass standard; lane 2-6, 24 h/48 h/72 h/96 h/120 h; B. SDS-PAGE analysis of 10 μL-aliquots of the IMAC purified codon-optimized GalOx gene with a C-terminal His_6_-tag; lane 1, molecular mass standard; lane 2, 1:10 dilution of GalOx-His_6_; lane 3, pure GalOx-His_6 _(2 mg·mL^-1^); C. Western blot analysis of 10 μL-aliquots of the IMAC purified codon-optimized GalOx gene with a C-terminal His_6_-tag; lane 1 and 2, Western blot of 1:10 dilution of GalOx-His_6 _and pure GalOx-His_6 _(2 mg·mL^-1^).

As shown in Figure 6, the amount of active GalOx obtained after 120 h of cultivation was very similar for all the three tested constructs. The codon-optimized gene unexpectedly did not result in an increased enzyme expression, which indicates that, although one in four nucleotides was changed from the wild-type (Additional File 1, Figure S13), codon usage does not limit translation. Regarding the OD_600_, the DCW and the protein content in the media, the three cultures showed very similar values of OD_600 _between 45-50, a DCW between 13.5-15.0 mg·mL^-1 ^and a protein content of 0.29-0.32 mg·mL^-1^. Incubation of the enzymes with 0.5 mM CuSO_4 _at 4°C overnight resulted in a 4-fold increase in activity for all 3 enzyme variants, thus indicating that the C-terminal His_6_-tag did not interfere with activation of GalOx by copper (II) ions. This was also supported by SDS-PAGE analysis of various samples taken during cultivation, each showing a distinct band at 65 kDa (Figure 7A).

A subsequent IMAC purification of the His_6_-tagged GalOx enzyme was performed, resulting in a concentrated enzyme preparation, which could be analyzed for the correct formation of the Tyr-Cys radical by visible spectroscopy (350-800 nm). A characteristic peak at 445 nm confirmed the formation of the radical cofactor [5,17]. The purified enzyme was also analyzed by SDS-PAGE and Western Blot (Figure 7B &7C), which indicated the presence of the correctly formed radical Tyr-Cys cofactor, the His_6_-tag at the C-terminus of the enzyme and a minimal amount of proteolytic degradation products. The high volumetric productivity of 580 U·L^-1^·h^-1 ^and possibility for simple purification by IMAC indicate that the extracellular expression of GalOx with a C-terminal His_6_-tag by *P. pastoris *is perhaps the best strategy for the large-scale production of this enzyme among the systems tested.

### Potential for further activation of GalOx by an inorganic oxidant

Solutions of produced GalOx are often a mixture of the fully active (Cu^2+^/Tyr-Cys^**·**^) and the inactive (Cu^2+^/Tyr-Cys) form [5,23]. Previous studies have indicated that treatment of GalOx enzyme preparations with the oxidizing agents, such as peroxidases or K_3_Fe(CN)_6_, results in the conversion of the inactive form into the fully active, radical species [5,14,17,23,24]. To test if the enzymes prepared in the present study could be further activated in the presence of an additional oxidizing agent, incubation of preparations with various concentrations of K_3_Fe(CN)_6 _and CuSO_4 _were performed. However, none of the experiments led to an additional increase in catalytic activity. Thus, the incubation of GalOx lacking the pro-peptide with 0.5 mM CuSO_4 _at 4°C under aerobic conditions overnight led to an efficient and apparently complete activation of the enzyme in all cases.

### Storage stability of extracellularly produced GalOx preparations from *P. pastoris*

To facilitate the direct use of GalOx preparations in applications where the presence of stabilizing additives (e.g., glycerol) may complicate the downstream isolation of carbohydrate products, simple storage conditions were explored. When stored at 4°C, the enzyme did not lose any activity for the tested period of 4 weeks. Snap-frozen (N_2(__*l*__)_), lyophilized samples exhibited a 50% decrease in catalytic activity. When GalOx was otherwise snap-frozen in liquid N_2_, stored in aliquots at -80°C and thawed on ice before measurements, no loss in catalytic activity or protein content was observed.

## Conclusions

In this study, codon-optimized and affinity tag variants of the *galox *gene from *F. graminearum *were examined for optimal heterologous intra- and extracellular production in *E. coli *and *P. pastoris*, two of the most easily accessible microbial expression hosts for general laboratory use. *E. coli*, in particular, is well-known as a fast-growing host, which can be readily transformed using a range of plasmid vectors. *E. coli *is thus the system of choice where both high transformation efficiency and high throughput are desired, such as in protein engineering studies via directed evolution [17,18]. Indeed, the codon-optimized M1 variant of GalOx, bearing an N-terminal His_10 _tag, is efficiently produced intracellularly in *E. coli *(Table 4), with both a good volumetric productivity (180 U·L^-1^·h^-1^) and high specific activity (180 U·mg^-1^, after IMAC purification and subsequent activation with Cu^++^).

The yeast *P. pastoris*, in contrast, has become one of the key eukaryotic expression hosts [27,28]. *P. pastoris *is transformed via chromosomal integration, with the drawback of a lower transformation efficiency than bacterial plasmid transformation, but with the advantage that stable genetic constructs are generated. The ability of *P. pastoris *to secrete proteins to the medium under the agency of the α-factor secretion signal peptide confers a significant advantage to this host, by greatly simplifying downstream protein isolation. Notably, the intracellular production of GalOx in *P. pastoris*, was plagued by a lower volumetric productivity (120 U·L^-1^·h^-1^) than in *E. coli *(Table 4), a greater amount of contaminating proteins, and the lack of a facile purification due to an intolerance for N-terminal oligo-histidine tagged constructs. In contrast, the extracellular production of the *F. graminearum *GalOx in this host was facile (volumetric productivity of 600 U·L^-1^·h^-1^, independent of yeast/fungal codon optimization, Table 4). A minimum amount of endogenous protein contaminants was observed, while the addition of a C-terminal His_6_-tag to GalOx provided a convenient strategy for removing colored medium components via IMAC. The observation that the enzyme could be directly activated to essentially similar levels by copper (II) ions added directly in the culture medium compared with post-harvest addition allows additional streamlining of GalOx production.

Thus, the intracellular *E. coli *and extracellular *P. pastoris *systems comprise a complementary pair for the production of GalOx, from high-throughput screening to production scale-up for applications.

## List of abbreviations used

DCW: dry cell weight; GalOx: galactose 6-oxidase; IMAC: immobilized metal affinity chromatography; IPTG: isopropyl β-D-1-thiogalactopyranoside; ABTS: 2,2'-azinobis(3-ethylbenzthiazolinesulfonic acid)]

## Competing interests

The authors declare that they have no competing interests.

## Authors' contributions

OS designed the experiments, performed the experiments and analyzed data, HB conceived the study and supervised research, LO aided experimental design and analysis, OS and HB wrote the paper. All authors read and approved the final manuscript.

## Supplementary Material

Additional file 1 **Sequences of galactose oxidase genes and primers**. Complete nucleotide and derived amino acid sequences of all galactoside oxidase constructs used in this study, including oligonucleotide primers used for cloning (13 figures and 1 table). Click here for file
