# Supplementary material for: A comparative summary of expression systems for the recombinant production of galactose oxidase
Source: Microb Cell Fact. 2010 Sep 13;9:68. doi: 10.1186/1475-2859-9-68 (PMC2949794; doi:10.1186/1475-2859-9-68)
Supplement: Additional file 1 — Sequences of galactose oxidase genes and primers. Complete nucleotide and derived amino acid sequences of all galactoside oxidase constructs used in this study, including oligonucleotide primers used for cloning (13 figures and 1 table). [file 1475-2859-9-68-S1.PDF]

Figure S1. Nucleotide and amino acid sequence of the *Fusarium* wt *galox* gene with an N-terminal His_{10}-tag in $pET16b^+$

atgggccatcatcatcatcatcatcatcatcatcacagcagcggccatatcgaaggtcgtcatatggcctcagcacctatcgg
aagcgccatttctcgcaacaactgggccgtcacttgcgacagtgcacagtcgggaaatgaatgcaacaaggccattgatggca
acaaggatacctttggcacacattctatggcgccaacggggatccaaagcccccctcacacatacacgattgacatgaagaca
actcagaacgtcaacggcttgtctatgctgcctcgacaggatggtaaccaaaacggctggatcggtcgccatgaggtttatct
aagctcagatggcacaaactggggcagccctgttgcgtcaggtagttggttcgccgactctactacaaaatactccaactttg
aaactcgccctgctcgctatgttcgtcttgtcgctatcactgaagcgaatggccagccttggactagcattgcagagatcaac
gtcttccaagctagttcttacacagcccccccagcctggtcttggacgctggggtccgactattgacttaccgattgttcctgc
ggctgcagcaattgaaccgacatcgggacgagtccttatgtggtcttcatatcgcaatgatgcatttggaggatcccctggtg
gtatcactttgacgtcttcctgggatccatccactggtattgtttccgaccgcactgtgacagtcaccaagcatgatatgttc
tgccctggtatctccatggatggtaacggtcagatcgtagtcacaggtggcaacgatgccaagaagaccagtttgtatgattc
atctagcgatagctggatcccgggacctgacatgcaagtggctcgtgggtatcagtcatcagctaccatgtcagacggtcgtg
tttttaccattggaggctcctggagcggtggcgtatttgagaagaatggcgaagtctatagcccatcttcaaagacatggacg
tccctacccaatgccaaggtcaacccaatgttgacggctgacaagcaaggattgtaccgttcagacaaccacgcgtggctctt
tggatggaagaagggttcggtgttccaagcgggacctagcacagccatgaactggtactataccagtggaagtggtgatgtga
agtcagccggaaaacgccagtctaaccgtggtgtagcccctgatgccatgtgcggaaacgctgtcatgtacgacgccgttaaa
ggaaagatcctgacctttggcggctccccagattatcaagactctgacgccacaaccaacgcccacatcatcaccctcggtga
acccggaacatctcccaacactgtctttgctagcaatgggttgtactttgcccgaacgtttcacacctctgttgttcttccag
acggaagcacgtttattacaggaggccaacgacgtggaattccgttcgaggattcaaccccggtatttacacctgagatctac
gtccctgaacaagacactttctacaagcagaaccccaactccattgttcgcgtctaccatagcatttccctttgttacctga
tggcagggtatttaacggtggtggtggtctttgtggcgattgtaccacgaatcatttcgacgcgcaaatctttacgccaaact
atctttacaatagcaacggcaatctcgcgacacgtcccaagattaccagaacctctacacagagcgtcaaggtcggtggcaga
attacaatctcgacggattcttcgattagcaaggcgtcgttgattcgctatggtacagcgacacacacggttaatactgacca
gcgccgcattcccctgactctgacaaacaatggaggaaatagctattctttccaagttcctagcgactctggtgttgctttgc
ctggctactggatgttgttcgtgatgaactcggccggtgttcctagtgtggcttcgacgattcgcgttactcagtga

MGHHHHHHHHHHSSGHIEGRHMASAPIGSAISRNNWAVTCDSAQSGNECNKAIDGNKDTFWHTFYGANGDPKPPHTYTIDMKT
TQNVNGLSMLPRQDGNQNGWIGRHEVYLSSDGTNWGSPVASGSWFADSTTKYSNFETRPARYVRLVAITEANGQPWTSIAEIN
VFQASSYTAPQPGLGRWGPTIDLPIVPAAAAIEPTSGRVLMWSSYRNDAFGGSPGGITLTSSWDPSTGIVSDRTVTVTKHDMF
CPGISMDGNGQIVVTGGNDAKKTSLYDSSSDSWIPGPDMQVARGYQSSATMSDGRVFTIGGSWSGGVFEKNGEVYSPSSKTWT
SLPNAKVNPMLTADKQGLYRSDNHAWLFGWKKGSVFQAGPSTAMNWYYTSGSGDVKSAGKRQSNRGVAPDAMCGNAVMYDAVK
GKILTFGGSPDYQDSDATTNAHIITLGEPGTSPNTVFASNGLYFARTFHTSVVLPDGSTFITGGQRRGIPFEDSTPVFTPEIY
VPEQDTFYKQNPNSIVRVYHSISLLLPDGRVFNGGGGLCGDCTTNHFDAQIFTPNYLYNSNGNLATRPKITRTSTQSVKVGGR
ITISTDSSISKASLIRYGTATHTVNTDQRRIPLTLTNNGGNSYSFQVPSDSGVALPGYWMLFVMNSAGVPSVASTIRVTQ-

Figure S2. Nucleotide and amino acid sequence of the M1 *galox* gene (optimized for *E.coli*) with an N-terminal His_{10}-tag in pET16b^{+}

```
atgggccatcatcatcatcatcatcatcatcatcacagcagcggccatatcgaaggtcgtcatatggcctccgcacctatcgg
tagcgccattcctcgcaacaactgggccgtcacttgcgacagtgcacagtcgggaaatgaatgcaacaaggccattgatggca
acaaggatacctttggcacacattctatggcgccaacggggatccaaagccccctcacacatacacgattgacatgaagaca
actcagaacgtcaacggcttgtctgtgctgcctcgacaggatggtaaccaaaacggctggatcggtcgccatgaggtttatct
aagctcagatggcacaaactggggcagccctgttgcgtcaggtagttggttcgccgactctactacaaaatactccaactttg
aaactcgccctgctcgctatgttcgtcttgtcgctatcactgaagcgaatggccagccctggactagcattgcagagatcaac
gtcttccaagctagttcttacacagcccccccagcctggtcttggacgctggggtccgactattgacttaccgattgttcctgc
ggctgcagcaattgaaccgacatcgggacgagtccttatgtggtcttcatatcgcaatgatgcatttgaaggatcccctggtg
gtatcactttgacgtcttcctgggatccatccactggtattgtttccgaccgcactgtgacagtcaccaagcatgatatgttc
tgccctggtatctccatggatggtaacggtcagatcgtagtcacaggtggcaacgatgccaagaagaccagtttgtatgattc
atctagcgatagctggatcccgggacctgacatgcaagtggctcgtgggtatcagtcatcagctaccatgtcagacggtcgtg
tttttaccattggaggctcctggagcggtggcgtatttgagaagaatggcgaagtctatagcccatcttcaaagacatggacg
tccctacccaatgccaaggtcaacccaatgttgacggctgacaagcaaggattgtaccgttcagacaaccacgcgtggctctt
tggatggaagaagggttcggtgttccaagcgggacctagcacagccatgaactggtactataccagtggaagtggtgatgtga
agtcagccggaaaacgccagtctaaccgtggtgtagcccctgatgccatgtgcggaaacgctgtcatgtacgacgccgttaaa
ggaaagatcctgacctttggcggctccccagattatcaagactctgacgccacaaccaacgcccacatcatcaccctcggtga
acccggaacatctcccaacactgtctttgctagcaatgggttgtactttgcccgaacgtttcacacctctgttgttcttccag
acggaagcacgtttattacaggaggccaacgacgtggaattccgttcgaggattcaaccccggtatttacacctgagatctac
gtccctgaacaagacactttctacaagcagaaccccaactccattgttcgcgcctaccatagcatttccctttgttacctga
tggcagggtatttaacggtggtggtggtctttgtggcgattgtaccacgaatcatttcgacgcgcaaatctttacgccaaact
atctttacgatagcaacggcaatctcgcgacacgtcccaagattaccagaacctctacacagagcgtcaaggtcggtggcaga
attacaatctcgacggattcttcgattagcaaggcgtcgttgattcgctatggtacagcgacacacacggttaatactgacca
gcgccgcattcccctgactctgacaaacaatggaggaaatagctattctttccaagttcctagcgactctggtgttgctttgc
ctggctactggatgttgttcgtgatgaactcggccggtgttcctagtgtggcttcgacgattcgcgttactcagtga
```

```
MGHHHHHHHHHHSSGHIEGRHMASAPIGSAIPRNNWAVTCDSAQSGNECNKAIDGNKDTFWHTFYGANGDPKPPHTYTIDMKT
TQNVNGLSVLPRQDGNQNGWIGRHEVYLSSDGTNWGSPVASGSWFADSTTKYSNFETRPARYVRLVAITEANGQPWTSIAEIN
VFQASSYTAPQPGLGRWGPTIDLPIVPAAAAIEPTSGRVLMWSSYRNDAFEGSPGGITLTSSWDPSTGIVSDRTVTVTKHDMF
CPGISMDGNGQIVVTGGNDAKKTSLYDSSSDSWIPGPDMQVARGYQSSATMSDGRVFTIGGSWSGGVFEKNGEVYSPSSKTWT
SLPNAKVNPMLTADKQGLYRSDNHAWLFGWKKGSVFQAGPSTAMNWYYTSGSGDVKSAGKRQSNRGVAPDAMCGNAVMYDAVK
GKILTFGGSPDYQDSDATTNAHIITLGEPGTSPNTVFASNGLYFARTFHTSVVLPDGSTFITGGQRRGIPFEDSTPVFTPEIY
VPEQDTFYKQNPNSIVRAYHSISLLLPDGRVFNGGGGLCGDCTTNHFDAQIFTPNYLYDSNGNLATRPKITRTSTQSVKVGGR
ITISTDSSISKASLIRYGTATHTVNTDQRRIPLTLTNNGGNSYSFQVPSDSGVALPGYWMLFVMNSAGVPSVASTIRVTQ-
```

Figure S3. Nucleotide and amino acid sequence of the *Fusarium* wt *galox* gene without a His-tag in pPIC3.5

```
atggcctcagcacctatcggaagcgccatttctcgcaacaactgggccgtcacttgcgacagtgcacagtcgggaaatgaatg
caacaaggccattgatggcaacaaggatacctttggcacacattctatggcgccaacggggatccaaagccccctcacacat
acacgattgacatgaagacaactcagaacgtcaacggcttgtctatgctgcctcgacaggatggtaaccaaaacggctggatc
ggtcgccatgaggtttatctaagctcagatggcacaaactggggcagccctgttgcgtcaggtagttggttcgccgactctac
tacaaaatactccaactttgaaactcgccctgctcgctatgttcgtcttgtcgctatcactgaagcgaatggccagccttgga
ctagcattgcagagatcaacgtcttccaagctagttcttacacagccccccagcctggtcttggacgctggggtccgactatt
gacttaccgattgttcctgcggctgcagcaattgaaccgacatcgggacgagtccttatgtggtcttcatatcgcaatgatgc
atttggaggatcccctggtggtatcactttgacgtcttcctgggatccatccactggtattgtttccgaccgcactgtgacag
tcaccaagcatgatatgttctgccctggtatctccatggatggtaacggtcagatcgtagtcacaggtggcaacgatgccaag
aagaccagtttgtatgattcatctagcgatagctggatcccgggacctgacatgcaagtggctcgtgggtatcagtcatcagc
taccatgtcagacggtcgtgtttttaccattggaggctcctggagcggtggcgtatttgagaagaatggcgaagtctatagcc
catcttcaaagacatggacgtccctacccaatgccaaggtcaacccaatgttgacggctgacaagcaaggattgtaccgttca
gacaaccacgcgtggctctttggatggaagaagggttcggtgttccaagcgggacctagcacagccatgaactggtactatac
cagtggaagtggtgatgtgaagtcagccggaaaacgccagtctaaccgtggtgtagcccctgatgccatgtgcggaaacgctg
tcatgtacgacgccgttaaaggaaagatcctgacctttggcggctccccagattatcaagactctgacgccacaaccaacgcc
cacatcatcaccctcggtgaacccggaacatctcccaacactgtctttgctagcaatgggttgtactttgcccgaacgtttca
cacctctgttgttcttccagacggaagcacgtttattacaggaggccaacgacgtggaattccgttcgaggattcaaccccgg
tatttacacctgagatctacgtccctgaacaagacactttctacaagcagaaccccaactccattgttcgcgtctaccatagc
atttccctttttgttacctgatggcagggtatttaacggtggtggtggtctttgtggcgattgtaccacgaatcatttcgacgc
gcaaatctttacgccaaactatctttacaatagcaacggcaatctcgcgacacgtcccaagattaccagaacctctacacaga
gcgtcaaggtcggtggcagaattacaatctcgacggattcttcgattagcaaggcgtcgttgattcgctatggtacagcgaca
cacacggttaatactgaccagcgccgcattcccctgactctgacaaacaatggaggaaatagctattctttccaagttcctag
cgactctggtgttgctttgcctggctactggatgttgttcgtgatgaactcggccggtgttcctagtgtggcttcgacgattc
gcgttactcagtga
```

```
MASAPIGSAISRNNWAVTCDSAQSGNECNKAIDGNKDTFWHTFYGANGDPKPPHTYTIDMKTTQNVNGLSMLPRQDGNQNGWI
GRHEVYLSSDGTNWGSPVASGSWFADSTTKYSNFETRPARYVRLVAITEANGQPWTSIAEINVFQASSYTAPQPGLGRWGPTI
DLPIVPAAAAIEPTSGRVLMWSSYRNDAFGGSPGGITLTSSWDPSTGIVSDRTVTVTKHDMFCPGISMDGNGQIVVTGGNDAK
KTSLYDSSSDSWIPGPDMQVARGYQSSATMSDGRVFTIGGSWSGGVFEKNGEVYSPSSKTWTSLPNAKVNPMLTADKQGLYRS
DNHAWLFGWKKGSVFQAGPSTAMNWYYTSGSGDVKSAGKRQSNRGVAPDAMCGNAVMYDAVKGKILTFGGSPDYQDSDATTNA
HIITLGEPGTSPNTVFASNGLYFARTFHTSVVLPDGSTFITGGQRRGIPFEDSTPVFTPEIYVPEQDTFYKQNPNSIVRVYHS
ISLLLPDGRVFNGGGGLCGDCTTNHFDAQIFTPNYLYNSNGNLATRPKITRTSTQSVKVGGRITISTDSSISKASLIRYGTAT
HTVNTDQRRIPLTLTNNGGNSYSFQVPSDSGVALPGYWMLFVMNSAGVPSVASTIRVTQ-
```

Figure S4. Nucleotide and amino acid sequence of the M1 *galox* gene (optimized for *E.coli*) without a His-tag in pPIC3.5

```
atggcctccgcacctatcggtagcgccattcctcgcaacaactgggccgtcacttgcgacagtgcacagtcgggaaatgaatg
caacaaggccattgatggcaacaaggatacctttggcacacattctatggcgccaacggggatccaaagccccctcacacat
acacgattgacatgaagacaactcagaacgtcaacggcttgtctgtgctgcctcgacaggatggtaaccaaaacggctggatc
ggtcgccatgaggtttatctaagctcagatggcacaaactggggcagccctgttgcgtcaggtagttggttcgccgactctac
tacaaaatactccaactttgaaactcgccctgctcgctatgttcgtcttgtcgctatcactgaagcgaatggccagccctgga
ctagcattgcagagatcaacgtcttccaagctagttcttacacagccccccagcctggtcttggacgctggggtccgactatt
gacttaccgattgttcctgcggctgcagcaattgaaccgacatcgggacgagtccttatgtggtcttcatatcgcaatgatgc
atttgaaggatcccctggtggtatcactttgacgtcttcctgggatccatccactggtattgtttccgaccgcactgtgacag
tcaccaagcatgatatgttctgccctggtatctccatggatggtaacggtcagatcgtagtcacaggtggcaacgatgccaag
aagaccagtttgtatgattcatctagcgatagctggatcccgggacctgacatgcaagtggctcgtgggtatcagtcatcagc
taccatgtcagacggtcgtgtttttaccattggaggctcctggagcggtggcgtatttgagaagaatggcgaagtctatagcc
catcttcaaagacatggacgtccctacccaatgccaaggtcaacccaatgttgacggctgacaagcaaggattgtaccgttca
gacaaccacgcgtggctctttggatggaagaagggttcggtgttccaagcgggacctagcacagccatgaactggtactatac
cagtggaagtggtgatgtgaagtcagccggaaaacgccagtctaaccgtggtgtagcccctgatgccatgtgcggaaacgctg
tcatgtacgacgccgttaaaggaaagatcctgacctttggcggctccccagattatcaagactctgacgccacaaccaacgcc
cacatcatcaccctcggtgaacccggaacatctcccaacactgtctttgctagcaatgggttgtactttgcccgaacgtttca
cacctctgttgttcttccagacggaagcacgtttattacaggaggccaacgacgtggaattccgttcgaggattcaaccccgg
tatttacacctgagatctacgtccctgaacaagacactttctacaagcagaaccccaactccattgttcgcgcctaccatagc
atttccctttttgttacctgatggcagggtatttaacggtggtggtggtctttgtggcgattgtaccacgaatcatttcgacgc
gcaaatctttacgccaaactatctttacgatagcaacggcaatctcgcgacacgtcccaagattaccagaacctctacacaga
gcgtcaaggtcggtggcagaattacaatctcgacggattcttcgattagcaaggcgtcgttgattcgctatggtacagcgaca
cacacggttaatactgaccagcgccgcattcccctgactctgacaaacaatggaggaaatagctattctttccaagttcctag
cgactctggtgttgctttgcctggctactggatgttgttcgtgatgaactcggccggtgttcctagtgtggcttcgacgattc
gcgttactcagtga
```

```
MASAPIGSAIPRNNWAVTCDSAQSGNECNKAIDGNKDTFWHTFYGANGDPKPPHTYTIDMKTTQNVNGLSVLPRQDGNQNGWI
GRHEVYLSSDGTNWGSPVASGSWFADSTTKYSNFETRPARYVRLVAITEANGQPWTSIAEINVFQASSYTAPQPGLGRWGPTI
DLPIVPAAAAIEPTSGRVLMWSSYRNDAFEGSPGGITLTSSWDPSTGIVSDRTVTVTKHDMFCPGISMDGNGQIVVTGGNDAK
KTSLYDSSSDSWIPGPDMQVARGYQSSATMSDGRVFTIGGSWSGGVFEKNGEVYSPSSKTWTSLPNAKVNPMLTADKQGLYRS
DNHAWLFGWKKGSVFQAGPSTAMNWYYTSGSGDVKSAGKRQSNRGVAPDAMCGNAVMYDAVKGKILTFGGSPDYQDSDATTNA
HIITLGEPGTSPNTVFASNGLYFARTFHTSVVLPDGSTFITGGQRRGIPFEDSTPVFTPEIYVPEQDTFYKQNPNSIVRAYHS
ISLLLPDGRVFNGGGGLCGDCTTNHFDAQIFTPNYLYDSNGNLATRPKITRTSTQSVKVGGRITISTDSSISKASLIRYGTAT
HTVNTDQRRIPLTLTNNGGNSYSFQVPSDSGVALPGYWMLFVMNSAGVPSVASTIRVTQ-
```

Figure S5. Nucleotide and amino acid sequence of the *Fusarium* wt *galox* gene with an N-terminal His_{10}-tag in pPIC3.5

```
atgggccatcatcatcatcatcatcatcatcatcacagcagcggccatatcgaaggtcgtcatatggcctcagcacctatcgg
aagcgccatttctcgcaacaactgggccgtcacttgcgacagtgcacagtcgggaaatgaatgcaacaaggccattgatggca
acaaggataccttttggcacacattctatggcgccaacggggatccaaagccccctcacacatacacgattgacatgaagaca
actcagaacgtcaacggcttgtctatgctgcctcgacaggatggtaaccaaaacggctggatcggtcgccatgaggtttatct
aagctcagatggcacaaactggggcagccctgttgcgtcaggtagttggttcgccgactctactacaaaatactccaactttg
aaactcgccctgctcgctatgttcgtcttgtcgctatcactgaagcgaatggccagccttggactagcattgcagagatcaac
gtcttccaagctagttcttacacagcccccagcctggtcttggacgctggggtccgactattgacttaccgattgttcctgc
ggctgcagcaattgaaccgacatcgggacgagtccttatgtggtcttcatatcgcaatgatgcatttggaggatcccctggtg
gtatcactttgacgtcttcctgggatccatccactggtattgtttccgaccgcactgtgacagtcaccaagcatgatatgttc
tgccctggtatctccatggatggtaacggtcagatcgtagtcacaggtggcaacgatgccaagaagaccagtttgtatgattc
atctagcgatagctggatcccgggacctgacatgcaagtggctcgtgggtatcagtcatcagctaccatgtcagacggtcgtg
tttttaccattggaggctcctggagcggtggcgtatttgagaagaatggcgaagtctatagcccatcttcaaagacatggacg
tccctacccaatgccaaggtcaacccaatgttgacggctgacaagcaaggattgtaccgttcagacaaccacgcgtggctctt
tggatggaagaagggttcggtgttccaagcgggacctagcacagccatgaactggtactataccagtggaagtggtgatgtga
agtcagccggaaaacgccagtctaaccgtggtgtagcccctgatgccatgtgcggaaacgctgtcatgtacgacgccgttaaa
ggaaagatcctgacctttggcggctccccagattatcaagactctgacgccacaaccaacgcccacatcatcaccctcggtga
acccggaacatctcccaacactgtctttgctagcaatgggttgtactttgcccgaacgtttcacacctctgttgttcttccag
acggaagcacgtttattacaggaggccaacgacgtggaattccgttcgaggattcaaccccggtatttacacctgagatctac
gtccctgaacaagacactttctacaagcagaaccccaactccattgttcgcgtctaccatagcatttccctttgttacctga
tggcagggtatttaacggtggtggtggtctttgtggcgattgtaccacgaatcatttcgacgcgcaaatctttacgccaaact
atctttacaatagcaacggcaatctcgcgacacgtcccaagattaccagaacctctacacagagcgtcaaggtcggtggcaga
attacaatctcgacggattcttcgattagcaaggcgtcgttgattcgctatggtacagcgacacacacggttaatactgacca
gcgccgcattcccctgactctgacaaacaatggaggaaatagctattctttccaagttcctagcgactctggtgttgctttgc
ctggctactggatgttgttcgtgatgaactcggccggtgttcctagtgtggcttcgacgattcgcgttactcagtga
```

```
MGHHHHHHHHHHSSGHIEGRHMASAPIGSAISRNNWAVTCDSAQSGNECNKAIDGNKDTFWHTFYGANGDPKPPHTYTIDMKT
TQNVNGLSMLPRQDGNQNGWIGRHEVYLSSDGTNWGSPVASGSWFADSTTKYSNFETRPARYVRLVAITEANGQPWTSIAEIN
VFQASSYTAPQPGLGRWGPTIDLPIVPAAAAIEPTSGRVLMWSSYRNDAFGGSPGGITLTSSWDPSTGIVSDRTVTVTKHDMF
CPGISMDGNGQIVVTGGNDAKKTSLYDSSSDSWIPGPDMQVARGYQSSATMSDGRVFTIGGSWSGGVFEKNGEVYSPSSKTWT
SLPNAKVNPMLTADKQGLYRSDNHAWLFGWKKGSVFQAGPSTAMNWYYTSGSGDVKSAGKRQSNRGVAPDAMCGNAVMYDAVK
GKILTFGGSPDYQDSDATTNAHIITLGEPGTSPNTVFASNGLYFARTFHTSVVLPDGSTFITGGQRRGIPFEDSTPVFTPEIY
VPEQDTFYKQNPNSIVRVYHSISLLLPDGRVFNGGGGLCGDCTTNHFDAQIFTPNYLYNSNGNLATRPKITRTSTQSVKVGGR
ITISTDSSISKASLIRYGTATHTVNTDQRRIPLTLTNNGGNSYSFQVPSDSGVALPGYWMLFVMNSAGVPSVASTIRVTQ-
```

Figure S6. Nucleotide and amino acid sequence of the M1 *galox* gene (optimized for *E.coli*) with an N-terminal His_{10}-tag in pPIC3.5

atgggccatcatcatcatcatcatcatcatcatcacagcagcggccatatcgaaggtcgtcatatggcctccgcacctatcgg
tagcgccattcctcgcaacaactgggccgtcacttgcgacagtgcacagtcgggaaatgaatgcaacaaggccattgatggca
acaaggataccttttggcacacattctatggcgccaacggggatccaaagccccctcacacatacacgattgacatgaagaca
actcagaacgtcaacggcttgtctgtgctgcctcgacaggatggtaaccaaaacggctggatcggtcgccatgaggtttatct
aagctcagatggcacaaactggggcagccctgttgcgtcaggtagttggttcgccgactctactacaaaatactccaactttg
aaactcgccctgctcgctatgttcgtcttgtcgctatcactgaagcgaatggccagccctggactagcattgcagagatcaac
gtcttccaagctagttcttacacagcccccagcctggtcttggacgctggggtccgactattgacttaccgattgttcctgc
ggctgcagcaattgaaccgacatcgggacgagtccttatgtggtcttcatatcgcaatgatgcatttgaaggatcccctggtg
gtatcactttgacgtcttcctgggatccatccactggtattgtttccgaccgcactgtgacagtcaccaagcatgatatgttc
tgccctggtatctccatggatggtaacggtcagatcgtagtcacaggtggcaacgatgccaagaagaccagtttgtatgattc
atctagcgatagctggatcccgggacctgacatgcaagtggctcgtgggtatcagtcatcagctaccatgtcagacggtcgtg
tttttaccattggaggctcctggagcggtggcgtatttgagaagaatggcgaagtctatagcccatcttcaaagacatggacg
tccctacccaatgccaaggtcaacccaatgttgacggctgacaagcaaggattgtaccgttcagacaaccacgcgtggctctt
tggatggaagaagggttcggtgttccaagcgggacctagcacagccatgaactggtactataccagtggaagtggtgatgtga
agtcagccggaaaacgccagtctaaccgtggtgtagcccctgatgccatgtgcggaaacgctgtcatgtacgacgccgttaaa
ggaaagatcctgacctttggcggctccccagattatcaagactctgacgccacaaccaacgcccacatcatcaccctcggtga
acccggaacatctcccaacactgtctttgctagcaatgggttgtactttgcccgaacgtttcacacctctgttgttcttccag
acggaagcacgtttattacaggaggccaacgacgtggaattccgttcgaggattcaaccccggtatttacacctgagatctac
gtccctgaacaagacactttctacaagcagaaccccaactccattgttcgcgcctaccatagcatttccctttgttacctga
tggcagggtatttaacggtggtggtggtctttgtggcgattgtaccacgaatcatttcgacgcgcaaatctttacgccaaact
atctttacgatagcaacggcaatctcgcgacacgtcccaagattaccagaacctctacacagagcgtcaaggtcggtggcaga
attacaatctcgacggattcttcgattagcaaggcgtcgttgattcgctatggtacagcgacacacacggttaatactgacca
gcgccgcattcccctgactctgacaaacaatggaggaaatagctattctttccaagttcctagcgactctggtgttgctttgc
ctggctactggatgttgttcgtgatgaactcggccggtgttcctagtgtggcttcgacgattcgcgttactcagtga

MGHHHHHHHHHHSSGHIEGRHMASAPIGSAIPRNNWAVTCDSAQSGNECNKAIDGNKDTFWHTFYGANGDPKPPHTYTIDMKT
TQNVNGLSVLPRQDGNQNGWIGRHEVYLSSDGTNWGSPVASGSWFADSTTKYSNFETRPARYVRLVAITEANGQPWTSIAEIN
VFQASSYTAPQPGLGRWGPTIDLPIVPAAAAIEPTSGRVLMWSSYRNDAFEGSPGGITLTSSWDPSTGIVSDRTVTVTKHDMF
CPGISMDGNGQIVVTGGNDAKKTSLYDSSSDSWIPGPDMQVARGYQSSATMSDGRVFTIGGSWSGGVFEKNGEVYSPSSKTWT
SLPNAKVNPMLTADKQGLYRSDNHAWLFGWKKGSVFQAGPSTAMNWYYTSGSGDVKSAGKRQSNRGVAPDAMCGNAVMYDAVK
GKILTFGGSPDYQDSDATTNAHIITLGEPGTSPNTVFASNGLYFARTFHTSVVLPDGSTFITGGQRRGIPFEDSTPVFTPEIY
VPEQDTFYKQNPNSIVRAYHSISLLLPDGRVFNGGGGLCGDCTTNHFDAQIFTPNYLYDSNGNLATRPKITRTSTQSVKVGGR
ITISTDSSISKASLIRYGTATHTVNTDQRRIPLTLTNNGGNSYSFQVPSDSGVALPGYWMLFVMNSAGVPSVASTIRVTQ-

Figure S7. Nucleotide and amino acid sequence of the *Fusarium* wt *galox* gene without a His-tag in pPICZα-C

atgagatttccttcaatttttactgctgttttattcgcagcatcctccgcattagctgctccagtcaacactacaacagaaga
tgaaacggcacaaattccggctgaagctgtcatcggttactcagatttagaaggggatttcgatgttgctgttttgccatttt
ccaacagcacaaataacgggttattgtttataaatactactattgccagcattgctgctaaagaagaaggggtatctctcgag
aagagagaggctgaagcatcgatgaattcacgtggcccagccggccgtctcggatcggtacctcgagccgcggcggccgccat
ggcctcagcacctatcggaagcgccatttctcgcaacaactgggccgtcacttgcgacagtgcacagtcgggaaatgaatgca
acaaggccattgatggcaacaaggataccttttggcacacattctatggcgccaacggggatccaaagccccctcacacatac
acgattgacatgaagacaactcagaacgtcaacggcttgtctatgctgcctcgacaggatggtaaccaaaacggctggatcgg
tcgccatgaggtttatctaagctcagatggcacaaactggggcagccctgttgcgtcaggtagttggttcgccgactctacta
caaaatactccaactttgaaactcgccctgctcgctatgttcgtcttgtcgctatcactgaagcgaatggccagccttggact
agcattgcagagatcaacgtcttccaagctagttcttacacagcccccagcctggtcttggacgctggggtccgactattga
cttaccgattgttcctgcggctgcagcaattgaaccgacatcgggacgagtccttatgtggtcttcatatcgcaatgatgcat
ttggaggatcccctggtggtatcactttgacgtcttcctgggatccatccactggtattgtttccgaccgcactgtgacagtc
accaagcatgatatgttctgccctggtatctccatggatggtaacggtcagatcgtagtcacaggtggcaacgatgccaagaa
gaccagtttgtatgattcatctagcgatagctggatcccgggacctgacatgcaagtggctcgtgggtatcagtcatcagcta
ccatgtcagacggtcgtgtttttaccattggaggctcctggagcggtggcgtatttgagaagaatggcgaagtctatagccca
tcttcaaagacatggacgtccctacccaatgccaaggtcaacccaatgttgacggctgacaagcaaggattgtaccgttcaga
caaccacgcgtggctctttggatggaagaagggttcggtgttccaagcgggacctagcacagccatgaactggtactatacca
gtggaagtggtgatgtgaagtcagccggaaaacgccagtctaaccgtggtgtagcccctgatgccatgtgcggaaacgctgtc
atgtacgacgccgttaaaggaaagatcctgacctttggcggctccccagattatcaagactctgacgccacaaccaacgccca
catcatcaccctcggtgaacccggaacatctcccaacactgtctttgctagcaatgggttgtactttgcccgaacgtttcaca
cctctgttgttcttccagacggaagcacgtttattacaggaggccaacgacgtggaattccgttcgaggattcaaccccggta
tttacacctgagatctacgtccctgaacaagacactttctacaagcagaaccccaactccattgttcgcgtctaccatagcat
ttccctttttgttacctgatggcagggtatttaacggtggtggtggtctttgtggcgattgtaccacgaatcatttcgacgcgc
aaatctttacgccaaactatctttacaatagcaacggcaatctcgcgacacgtcccaagattaccagaacctctacacagagc
gtcaaggtcggtggcagaattacaatctcgacggattcttcgattagcaaggcgtcgttgattcgctatggtacagcgacaca
cacggttaatactgaccagcgccgcattcccctgactctgacaaacaatggaggaaatagctattctttccaagttcctagcg
actctggtgttgctttgcctggctactggatgttgttcgtgatgaactcggccggtgttcctagtgtggcttcgacgattcgc
gttactcagtga

MRFPSIFTAVLFAASSALAAPVNTTTEDETAQIPAEAVIGYSDLEGDFDVAVLPFSNSTNNGLLFINTTIASIAAKEEGVSLE
KREAEASMNSRGPAGRLGSVPRAAAAAMASAPIGSAISRNNWAVTCDSAQSGNECNKAIDGNKDTFWHTFYGANGDPKPPHTY
TIDMKTTQNVNGLSMLPRQDGNQNGWIGRHEVYLSSDGTNWGSPVASGSWFADSTTKYSNFETRPARYVRLVAITEANGQPWT
SIAEINVFQASSYTAPQPGLGRWGPTIDLPIVPAAAAIEPTSGRVLMWSSYRNDAFGGSPGGITLTSSWDPSTGIVSDRTVTV
TKHDMFCPGISMDGNGQIVVTGGNDAKKTSLYDSSSDSWIPGPDMQVARGYQSSATMSDGRVFTIGGSWSGGVFEKNGEVYSP
SSKTWTSLPNAKVNPMLTADKQGLYRSDNHAWLFGWKKGSVFQAGPSTAMNWYYTSGSGDVKSAGKRQSNRGVAPDAMCGNAV
MYDAVKGKILTFGGSPDYQDSDATTNAHIITLGEPGTSPNTVFASNGLYFARTFHTSVVLPDGSTFITGGQRRGIPFEDSTPV
FTPEIYVPEQDTFYKQNPNSIVRVYHSISLLLPDGRVFNGGGGLCGDCTTNHFDAQIFTPNYLYNSNGNLATRPKITRTSTQS
VKVGGRITISTDSSISKASLIRYGTATHTVNTDQRRIPLTLTNNGGNSYSFQVPSDSGVALPGYWMLFVMNSAGVPSVASTIR
VTQ-

Figure S8. Nucleotide and amino acid sequence of the of the M1 *galox* gene (optimized for *E.coli*) without a His-tag in pPICZα-C

atgagatttccttcaatttttactgctgttttattcgcagcatcctccgcattagctgctccagtcaacactacaacagaaga
tgaaacggcacaaattccggctgaagctgtcatcggttactcagatttagaaggggatttcgatgttgctgttttgccatttt
ccaacagcacaaataacgggttattgtttataaatactactattgccagcattgctgctaaagaagaaggggtatctctcgag
aagagagaggctgaagcatcgatgaattcacgtggcccagccggccgtctcggatcggtacctcgagccgcggcggccgccat
ggcctccgcacctatcggtagcgccattcctcgcaacaactgggccgtcacttgcgacagtgcacagtcgggaaatgaatgca
acaaggccattgatggcaacaaggatacctttggcacacattctatggcgccaacggggatccaaagccccctcacacatac
acgattgacatgaagacaactcagaacgtcaacggcttgtctgtgctgcctcgacaggatggtaaccaaaacggctggatcgg
tcgccatgaggtttatctaagctcagatggcacaaactggggcagccctgttgcgtcaggtagttggttcgccgactctacta
caaaatactccaactttgaaactcgccctgctcgctatgttcgtcttgtcgctatcactgaagcgaatggccagccctggact
agcattgcagagatcaacgtcttccaagctagttcttacacagcccccagcctggtcttggacgctggggtccgactattga
cttaccgattgttcctgcggctgcagcaattgaaccgacatcgggacgagtccttatgtggtcttcatatcgcaatgatgcat
ttgaaggatcccctggtggtatcactttgacgtcttcctgggatccatccactggtattgtttccgaccgcactgtgacagtc
accaagcatgatatgttctgccctggtatctccatggatggtaacggtcagatcgtagtcacaggtggcaacgatgccaagaa
gaccagtttgtatgattcatctagcgatagctggatcccgggacctgacatgcaagtggctcgtgggtatcagtcatcagcta
ccatgtcagacggtcgtgtttttaccattggaggctcctggagcggtggcgtatttgagaagaatggcgaagtctatagccca
tcttcaaagacatggacgtccctacccaatgccaaggtcaacccaatgttgacggctgacaagcaaggattgtaccgttcaga
caaccacgcgtggctctttggatggaagaagggttcggtgttccaagcgggacctagcacagccatgaactggtactatacca
gtggaagtggtgatgtgaagtcagccggaaaacgccagtctaaccgtggtgtagcccctgatgccatgtgcggaaacgctgtc
atgtacgacgccgttaaaggaaagatcctgacctttggcggctccccagattatcaagactctgacgccacaaccaacgccca
catcatcaccctcggtgaacccggaacatctcccaacactgtctttgctagcaatgggttgtactttgcccgaacgtttcaca
cctctgttgttcttccagacggaagcacgtttattacaggaggccaacgacgtggaattccgttcgaggattcaaccccggta
tttacacctgagatctacgtccctgaacaagacactttctacaagcagaaccccaactccattgttcgcgcctaccatagcat
ttccctttttgttacctgatggcagggtatttaacggtggtggtggtctttgtggcgattgtaccacgaatcatttcgacgcgc
aaatctttacgccaaactatctttacgatagcaacggcaatctcgcgacacgtcccaagattaccagaacctctacacagagc
gtcaaggtcggtggcagaattacaatctcgacggattcttcgattagcaaggcgtcgttgattcgctatggtacagcgacaca
cacggttaatactgaccagcgccgcattcccctgactctgacaaacaatggaggaaatagctattctttccaagttcctagcg
actctggtgttgctttgcctggctactggatgttgtcgtgatgaactcggccggtgttcctagtgtggcttcgacgattcgcg
ttactcagtga

MRFPSIFTAVLFAASSALAAPVNTTTEDETAQIPAEAVIGYSDLEGDFDVAVLPFSNSTNNGLLFINTTIASIAAKEEGVSLE
KREAEASMNSRGPAGRLGSVPRAAAAAMASAPIGSAIPRNNWAVTCDSAQSGNECNKAIDGNKDTFWHTFYGANGDPKPPHTY
TIDMKTTQNVNGLSVLPRQDGNQNGWIGRHEVYLSSDGTNWGSPVASGSWFADSTTKYSNFETRPARYVRLVAITEANGQPWT
SIAEINVFQASSYTAPQPGLGRWGPTIDLPIVPAAAAIEPTSGRVLMWSSYRNDAFEGSPGGITLTSSWDPSTGIVSDRTVTV
TKHDMFCPGISMDGNGQIVVTGGNDAKKTSLYDSSSDSWIPGPDMQVARGYQSSATMSDGRVFTIGGSWSGGVFEKNGEVYSP
SSKTWTSLPNAKVNPMLTADKQGLYRSDNHAWLFGWKKGSVFQAGPSTAMNWYYTSGSGDVKSAGKRQSNRGVAPDAMCGNAV
MYDAVKGKILTFGGSPDYQDSDATTNAHIITLGEPGTSPNTVFASNGLYFARTFHTSVVLPDGSTFITGGQRRGIPFEDSTPV
FTPEIYVPEQDTFYKQNPNSIVRAYHSISLLLPDGRVFNGGGGLCGDCTTNHFDAQIFTPNYLYDSNGNLATRPKITRTSTQS
VKVGGRITISTDSSISKASLIRYGTATHTVNTDQRRIPLTLTNNGGNSYSFQVPSDSGVALPGYWMLFVMNSAGVPSVASTIR
VTQ-

Figure S9. Nucleotide and amino acid sequence of the *Fusarium* wt *galox* gene with an N-terminal His_{10}-tag in pPICZα-C

atgagatttccttcaatttttactgctgttttattcgcagcatcctccgcattagctgctccagtcaacactacaacagaaga
tgaaacggcacaaattccggctgaagctgtcatcggttactcagatttagaaggggatttcgatgttgctgttttgccatttt
ccaacagcacaaataacgggttattgtttataaatactactattgccagcattgctgctaaagaagaaggggtatctctcgag
aagagagaggctgaagcatcgatgaattcacgtggcccagccggccgtctcggatcggtacctcgagccgcggcggccgccat
gggccatcatcatcatcatcatcatcatcatcacagcagcggccatatcgaaggtcgtcatatggcctcagcacctatcggaa
gcgccatttctcgcaacaactgggccgtcacttgcgacagtgcacagtcgggaaatgaatgcaacaaggccattgatggcaac
aaggatacctttggcacacattctatggcgccaacggggatccaaagcccctcacacatacacgattgacatgaagacaac
tcagaacgtcaacggcttgtctatgctgcctcgacaggatggtaaccaaaacggctggatcggtcgccatgaggtttatctaa
gctcagatggcacaaactggggcagccctgttgcgtcaggtagttggttcgccgactctactacaaaatactccaactttgaa
actcgccctgctcgctatgttcgtcttgtcgctatcactgaagcgaatggccagccttggactagcattgcagagatcaacgt
cttccaagctagttcttacacagcccccagcctggtcttggacgctggggtccgactattgacttaccgattgttcctgcgg
ctgcagcaattgaaccgacatcgggacgagtccttatgtggtcttcatatcgcaatgatgcatttggaggatcccctggtggt
atcactttgacgtcttcctgggatccatccactggtattgtttccgaccgcactgtgacagtcaccaagcatgatatgttctg
ccctggtatctccatggatggtaacggtcagatcgtagtcacaggtggcaacgatgccaagaagaccagtttgtatgattcat
ctagcgatagctggatcccgggacctgacatgcaagtggctcgtgggtatcagtcatcagctaccatgtcagacggtcgtgtt
tttaccattggaggctcctggagcggtggcgtatttgagaagaatggcgaagtctatagcccatcttcaaagacatggacgtc
cctacccaatgccaaggtcaacccaatgttgacggctgacaagcaaggattgtaccgttcagacaaccacgcgtggctctttg
gatggaagaagggttcggtgttccaagcgggacctagcacagccatgaactggtactataccagtggaagtggtgatgtgaag
tcagccggaaaacgccagtctaaccgtggtgtagcccctgatgccatgtgcggaaacgctgtcatgtacgacgccgttaaagg
aaagatcctgacctttggcggctccccagattatcaagactctgacgccacaaccaacgcccacatcatcaccctcggtgaac
ccggaacatctcccaacactgtctttgctagcaatgggttgtactttgcccgaacgtttcacacctctgttgttcttccagac
ggaagcacgtttattacaggaggccaacgacgtggaattccgttcgaggattcaaccccggtatttacacctgagatctacgt
ccctgaacaagacactttctacaagcagaaccccaactccattgttcgcgtctaccatagcatttccctttttgttacctgatg
gcagggtatttaacggtggtggtggtctttgtggcgattgtaccacgaatcatttcgacgcgcaaatctttacgccaaactat
ctttacaatagcaacggcaatctcgcgacacgtcccaagattaccagaacctctacacagagcgtcaaggtcggtggcagaat
tacaatctcgacggattcttcgattagcaaggcgtcgttgattcgctatggtacagcgacacacacggttaatactgaccagc
gccgcattcccctgactctgacaaacaatggaggaaatagctattctttccaagttcctagcgactctggtgttgctttgcct
ggctactggatgttgttcgtgatgaactcggccggtgttcctagtgtggcttcgacgattcgcgttactcagtga

MRFPSIFTAVLFAASSALAAPVNTTTEDETAQIPAEAVIGYSDLEGDFDVAVLPFSNSTNNGLLFINTTIASIAAKEEGVSLE
KREAEASMNSRGPAGRLGSVPRAAAAAMGHHHHHHHHHHSSGHIEGRHMASAPIGSAISRNNWAVTCDSAQSGNECNKAIDGN
KDTFWHTFYGANGDPKPPHTYTIDMKTTQNVNGLSMLPRQDGNQNGWIGRHEVYLSSDGTNWGSPVASGSWFADSTTKYSNFE
TRPARYVRLVAITEANGQPWTSIAEINVFQASSYTAPQPGLGRWGPTIDLPIVPAAAAIEPTSGRVLMWSSYRNDAFGGSPGG
ITLTSSWDPSTGIVSDRTVTVTKHDMFCPGISMDGNGQIVVTGGNDAKKTSLYDSSSDSWIPGPDMQVARGYQSSATMSDGRV
FTIGGSWSGGVFEKNGEVYSPSSKTWTSLPNAKVNPMLTADKQGLYRSDNHAWLFGWKKGSVFQAGPSTAMNWYYTSGSGDVK
SAGKRQSNRGVAPDAMCGNAVMYDAVKGKILTFGGSPDYQDSDATTNAHIITLGEPGTSPNTVFASNGLYFARTFHTSVVLPD
GSTFITGGQRRGIPFEDSTPVFTPEIYVPEQDTFYKQNPNSIVRVYHSISLLLPDGRVFNGGGGLCGDCTTNHFDAQIFTPNY
LYNSNGNLATRPKITRTSTQSVKVGGRITISTDSSISKASLIRYGTATHTVNTDQRRIPLTLTNNGGNSYSFQVPSDSGVALP
GYWMLFVMNSAGVPSVASTIRVTQ-

Figure S10. Nucleotide and amino acid sequence of the M1 *galox* gene (optimized for *E.coli*) with an N-terminal His_{10}-tag in pPICZα-C

atgagatttccttcaatttttactgctgttttattcgcagcatcctccgcattagctgctccagtcaacactacaacagaaga
tgaaacggcacaaattccggctgaagctgtcatcggttactcagatttagaaggggatttcgatgttgctgttttgccatttt
ccaacagcacaaataacgggttattgtttataaatactactattgccagcattgctgctaaagaagaaggggtatctctcgag
aagagagaggctgaagcatcgatgaattcacgtggcccagccggccgtctcggatcggtacctcgagccgcggcggccgccat
gggccatcatcatcatcatcatcatcatcatcacagcagcggccatatcgaaggtcgtcatatggcctccgcacctatcggta
gcgccattcctcgcaacaactgggccgtcacttgcgacagtgcacagtcgggaaatgaatgcaacaaggccattgatggcaac
aaggatacctttggcacacattctatggcgccaacggggatccaaagccccctcacacatacacgattgacatgaagacaac
tcagaacgtcaacggcttgtctgtgctgcctcgacaggatggtaaccaaaacggctggatcggtcgccatgaggtttatctaa
gctcagatggcacaaactggggcagccctgttgcgtcaggtagttggttcgccgactctactacaaaatactccaactttgaa
actcgccctgctcgctatgttcgtcttgtcgctatcactgaagcgaatggccagccctggactagcattgcagagatcaacgt
cttccaagctagttcttacacagcccccagcctggtcttggacgctggggtccgactattgacttaccgattgttcctgcgg
ctgcagcaattgaaccgacatcgggacgagtccttatgtggtcttcatatcgcaatgatgcatttgaaggatcccctggtggt
atcactttgacgtcttcctgggatccatccactggtattgtttccgaccgcactgtgacagtcaccaagcatgatatgttctg
ccctggtatctccatggatggtaacggtcagatcgtagtcacaggtggcaacgatgccaagaagaccagtttgtatgattcat
ctagcgatagctggatcccgggacctgacatgcaagtggctcgtgggtatcagtcatcagctaccatgtcagacggtcgtgtt
tttaccattggaggctcctggagcggtggcgtatttgagaagaatggcgaagtctatagcccatcttcaaagacatggacgtc
cctacccaatgccaaggtcaacccaatgttgacggctgacaagcaaggattgtaccgttcagacaaccacgcgtggctctttg
gatggaagaagggttcggtgttccaagcgggacctagcacagccatgaactggtactataccagtggaagtggtgatgtgaag
tcagccggaaaacgccagtctaaccgtggtgtagcccctgatgccatgtgcggaaacgctgtcatgtacgacgccgttaaagg
aaagatcctgacctttggcggctccccagattatcaagactctgacgccacaaccaacgcccacatcatcaccctcggtgaac
ccggaacatctcccaacactgtctttgctagcaatgggttgtactttgcccgaacgtttcacacctctgttgttcttccagac
ggaagcacgtttattacaggaggccaacgacgtggaattccgttcgaggattcaaccccggtatttacacctgagatctacgt
ccctgaacaagacactttctacaagcagaaccccaactccattgttcgcgcctaccatagcatttccctttttgttacctgatg
gcagggtatttaacggtggtggtggtctttgtggcgattgtaccacgaatcatttcgacgcgcaaatctttacgccaaactat
ctttacgatagcaacggcaatctcgcgacacgtcccaagattaccagaacctctacacagagcgtcaaggtcggtggcagaat
tacaatctcgacggattcttcgattagcaaggcgtcgttgattcgctatggtacagcgacacacacggttaatactgaccagc
gccgcattcccctgactctgacaaacaatggaggaaatagctattctttccaagttcctagcgactctggtgttgctttgcct
ggctactggatgttgttcgtgatgaactcggccggtgttcctagtgtggcttcgacgattcgcgttactcagtga

MRFPSIFTAVLFAASSALAAPVNTTTEDETAQIPAEAVIGYSDLEGDFDVAVLPFSNSTNNGLLFINTTIASIAAKEEGVSLE
KREAEASMNSRGPAGRLGSVPRAAAAAMGHHHHHHHHHHSSGHIEGRHMASAPIGSAIPRNNWAVTCDSAQSGNECNKAIDGN
KDTFWHTFYGANGDPKPPHTYTIDMKTTQNVNGLSVLPRQDGNQNGWIGRHEVYLSSDGTNWGSPVASGSWFADSTTKYSNFE
TRPARYVRLVAITEANGQPWTSIAEINVFQASSYTAPQPGLGRWGPTIDLPIVPAAAAIEPTSGRVLMWSSYRNDAFEGSPGG
ITLTSSWDPSTGIVSDRTVTVTKHDMFCPGISMDGNGQIVVTGGNDAKKTSLYDSSSDSWIPGPDMQVARGYQSSATMSDGRV
FTIGGSWSGGVFEKNGEVYSPSSKTWTSLPNAKVNPMLTADKQGLYRSDNHAWLFGWKKGSVFQAGPSTAMNWYYTSGSGDVK
SAGKRQSNRGVAPDAMCGNAVMYDAVKGKILTFGGSPDYQDSDATTNAHIITLGEPGTSPNTVFASNGLYFARTFHTSVVLPD
GSTFITGGQRRGIPFEDSTPVFTPEIYVPEQDTFYKQNPNSIVRAYHSISLLLPDGRVFNGGGGLCGDCTTNHFDAQIFTPNY
LYDSNGNLATRPKITRTSTQSVKVGGRITISTDSSISKASLIRYGTATHTVNTDQRRIPLTLTNNGGNSYSFQVPSDSGVALP
GYWMLFVMNSAGVPSVASTIRVTQ-

Figure S11. Nucleotide and amino acid sequence of the *galox* gene optimized for *P. pastoris* without a His-tag in pPICZα-C

atgagatttccttcaatttttactgctgttttattcgcagcatcctccgcattagctgctccagtcaacactacaacagaaga
tgaaacggcacaaattccggctgaagctgtcatcggttactcagatttagaaggggatttcgatgttgctgttttgccatttt
ccaacagcacaaataacgggttattgtttataaatactactattgccagcattgctgctaaagaagaaggggtatctctcgag
aagagagaggctgaagcatcgatgaattcacgtggcccagccggccgtctcggatcggtacctcgagccgcggcggccgccat
ggcttctgctccaattggttcagctatttccagaaacaattgggctgttacttgtgattccgctcaatctggtaatgaatgta
acaaggctattgatggaaacaaggacacattttggcatactttctacggtgctaacggagatccaaaaccacctcacacttat
acaattgacatgaagactacacagaacgttaatggtttgtctatgttgcctagacaagatggaaaccagaatggttggatcgg
aagacatgaagtttacttgtcttccgatggtactaattggggatctcctgttgcttcaggttcttggtttgctgactctacta
caaagtactccaacttcgagactagaccagctagatatgttagattggttgctattacagaagctaatggtcaaccttggact
tctattgctgagatcaacgtttttcaagcttcatcttacacagctccacagcctggtttgggaagatggggtccaactattga
tttgccaatcgttcctgctgctgctgctattgagcctacttctggaagagttttgatgtggtcctcatatagaaatgacgctt
ttggtggatccccaggtggaattactttgacatcttcctgggatccttcaactggtatcgtttctgacagaactgttacagtt
actaagcatgatatgttctgtccaggaatttctatggacggtaatggacaaatcgttgttacaggtggaaacgatgctaagaa
aacttcattgtacgattcatcttccgactcttggattccaggtcctgatatgcaagttgctagaggatatcagtcatctgcta
caatgtctgacggtagagtttttactatcggtggatcctggtcaggtggagttttcgaaaagaacggagaggtttactcccca
tcctcaaagacatggacttcattgccaaatgctaaagttaaccctatgttgactgctgataagcaaggtttgtatagatctga
caatcacgcttggttgtttggttggaagaaaggatcagttttccaggctggaccttctacagctatgaactggtactatactt
ctggttccggagatgttaagtccgctggtaaaagacaatcaaatagaggagttgctccagatgctatgtgtggtaacgctgtt
atgtacgacgctgttaagggaaaaattttgactttggtggatctcctgactatcaggattccgacgctactacaaatgctca
tattatcactttgggtgaaccaggaacatcccctaatactgtttttgcttcaaacggtttgtactttgctagaacattccaca
cttctgttgttttgccagatggttccacattcattactggtggacaaagaagaggaatcccttttgaggattcaacaccagtt
ttcactcctgaaatctacgttccagagcaagacactttctacaagcagaaccctaattctatcgttagagtttaccattcaat
ctctttgttgttgccagatggtagagttttcaacggtggaggtggattgtgtggagattgtactacaaatcactttgacgctc
aaattttcactccaaactacttgtacaactccaatggtaacttggctactagacctaaaatcacaagaacttctacacagtcc
gttaaggttggtggaagaattacaatctctactgattcttccatttcaaaggcttctttgatcagatacggtactgctacaca
cactgttaatactgaccaaagaagaatcccattgacattgactaacaatggtggaaactcctactcatttcaggttccatctg
attccggtgttgctttgcctggatattggatgttgttcgttatgaactctgctggtgttccttccgttgcttcaacaattaga
gttactcaataa

MRFPSIFTAVLFAASSALAAPVNTTTEDETAQIPAEAVIGYSDLEGDFDVAVLPFSNSTNNGLLFINTTIASIAAKEEGVSLE
KREAEASMNSRGPAGRLGSVPRAAAAAMASAPIGSAISRNNWAVTCDSAQSGNECNKAIDGNKDTFWHTFYGANGDPKPPHTY
TIDMKTTQNVNGLSMLPRQDGNQNGWIGRHEVYLSSDGTNWGSPVASGSWFADSTTKYSNFETRPARYVRLVAITEANGQPWT
SIAEINVFQASSYTAPQPGLGRWGPTIDLPIVPAAAAIEPTSGRVLMWSSYRNDAFGGSPGGITLTSSWDPSTGIVSDRTVTV
TKHDMFCPGISMDGNGQIVVTGGNDAKKTSLYDSSSDSWIPGPDMQVARGYQSSATMSDGRVFTIGGSWSGGVFEKNGEVYSP
SSKTWTSLPNAKVNPMLTADKQGLYRSDNHAWLFGWKKGSVFQAGPSTAMNWYYTSGSGDVKSAGKRQSNRGVAPDAMCGNAV
MYDAVKGKILTFGGSPDYQDSDATTNAHIITLGEPGTSPNTVFASNGLYFARTFHTSVVLPDGSTFITGGQRRGIPFEDSTPV
FTPEIYVPEQDTFYKQNPNSIVRVYHSISLLLPDGRVFNGGGGLCGDCTTNHFDAQIFTPNYLYNSNGNLATRPKITRTSTQS
VKVGGRITISTDSSISKASLIRYGTATHTVNTDQRRIPLTLTNNGGNSYSFQVPSDSGVALPGYWMLFVMNSAGVPSVASTIR
VTQ-

Figure S12. Nucleotide and amino acid sequence of the *galox* gene optimized for *P. pastoris* with a C-terminal His_6-tag in pPICZα-C

atgagatttccttcaatttttactgctgttttattcgcagcatcctccgcattagctgctccagtcaacactacaacagaaga
tgaaacggcacaaattccggctgaagctgtcatcggttactcagatttagaaggggatttcgatgttgctgttttgccatttt
ccaacagcacaaataacgggttattgtttataaatactactattgccagcattgctgctaaagaagaaggggtatctctcgag
aagagagaggctgaagcatcgatgaattcacgtggcccagccggccgtctcggatcggtacctcgagccgcggcggccgccat
ggcttctgctccaattggttcagctatttccagaaacaattgggctgttacttgtgattccgctcaatctggtaatgaatgta
acaaggctattgatggaaacaaggacacattttggcatactttctacggtgctaacggagatccaaaaccacctcacacttat
acaattgacatgaagactacacagaacgttaatggtttgtctatgttgcctagacaagatggaaaccagaatggttggatcgg
aagacatgaagtttacttgtcttccgatggtactaattggggatctcctgttgcttcaggttcttggtttgctgactctacta
caaagtactccaacttcgagactagaccagctagatatgttagattggttgctattacagaagctaatggtcaaccttggact
tctattgctgagatcaacgtttttcaagcttcatcttacacagctccacagcctggtttgggaagatggggtccaactattga
tttgccaatcgttcctgctgctgctgctattgagcctacttctggaagagttttgatgtggtcctcatatagaaatgacgctt
ttggtggatccccaggtggaattactttgacatcttcctgggatccttcaactggtatcgtttctgacagaactgttacagtt
actaagcatgatatgttctgtccaggaatttctatggacggtaatggacaaatcgttgttacaggtggaaacgatgctaagaa
aacttcattgtacgattcatcttccgactcttggattccaggtcctgatatgcaagttgctagaggatatcagtcatctgcta
caatgtctgacggtagagttttactatcggtggatcctggtcaggtggagttttcgaaaagaacggagaggtttactcccca
tcctcaaagacatggacttcattgccaaatgctaaagttaaccctatgttgactgctgataagcaaggtttgtatagatctga
caatcacgcttggttgtttggttggaagaaaggatcagttttccaggctggaccttctacagctatgaactggtactatactt
ctggttccggagatgttaagtccgctggtaaaagacaatcaaatagaggagttgctccagatgctatgtgtggtaacgctgtt
atgtacgacgctgttaagggaaaaattttgacttttggtggatctcctgactatcaggattccgacgctactacaaatgctca
tattatcactttgggtgaaccaggaacatcccctaatactgtttttgcttcaaacggtttgtactttgctagaacattccaca
cttctgttgttttgccagatggttccacattcattactggtggacaaagaagaggaatccctttttgaggattcaacaccagtt
ttcactcctgaaatctacgttccagagcaagacactttctacaagcagaaccctaattctatcgttagagtttaccattcaat
ctctttgttgttgccagatggtagagttttcaacggtggaggtggattgtgtggagattgtactacaaatcactttgacgctc
aaattttcactccaaactacttgtacaactccaatggtaacttggctactagacctaaaatcacaagaacttctacacagtcc
gttaaggttggtggaagaattacaatctctactgattcttccatttcaaaggcttctttgatcagatacggtactgctacaca
cactgttaatactgaccaaagaagaatcccattgacattgactaacaatggtggaaactcctactcatttcaggttccatctg
attccggtgttgctttgcctggatattggatgttgttcgttatgaactctgctggtgttccttccgttgcttcaacaattaga
gttactcatctagaacaaaaactcatctcagaagaggatctgaatagcgccgtcgaccatcatcatcatcatcattga

MRFPSIFTAVLFAASSALAAPVNTTTEDETAQIPAEAVIGYSDLEGDFDVAVLPFSNSTNNGLLFINTTIASIAAKEEGVSLE
KREAEASMNSRGPAGRLGSVPRAAAAAMASAPIGSAISRNNWAVTCDSAQSGNECNKAIDGNKDTFWHTFYGANGDPKPPHTY
TIDMKTTQNVNGLSMLPRQDGNQNGWIGRHEVYLSSDGTNWGSPVASGSWFADSTTKYSNFETRPARYVRLVAITEANGQPWT
SIAEINVFQASSYTAPQPGLGRWGPTIDLPIVPAAAAIEPTSGRVLMWSSYRNDAFGGSPGGITLTSSWDPSTGIVSDRTVTV
TKHDMFCPGISMDGNGQIVVTGGNDAKKTSLYDSSSDSWIPGPDMQVARGYQSSATMSDGRVFTIGGSWSGGVFEKNGEVYSP
SSKTWTSLPNAKVNPMLTADKQGLYRSDNHAWLFGWKKGSVFQAGPSTAMNWYYTSGSGDVKSAGKRQSNRGVAPDAMCGNAV
MYDAVKGKILTFGGSPDYQDSDATTNAHIITLGEPGTSPNTVFASNGLYFARTFHTSVVLPDGSTFITGGQRRGIPFEDSTPV
FTPEIYVPEQDTFYKQNPNSIVRVYHSISLLLPDGRVFNGGGGLCGDCTTNHFDAQIFTPNYLYNSNGNLATRPKITRTSTQS
VKVGGRITISTDSSISKASLIRYGTATHTVNTDQRRIPLTLTNNGGNSYSFQVPSDSGVALPGYWMLFVMNSAGVPSVASTIR
VTHLEQKLISEEDLNSAVDHHHHHH-

Figure S13. Alignment of the wild-type *galox* gene from *Fusarium graminearum* and the codon-optimized variant for the expression in *Pichia pastoris*

```
wtgalox         ATGGCCTCAGCACCTATCGGAAGCGCCATTTCTCGCAACAACTGGGCCGTCACTTGCGAC 60
optgalox        ATGGCTTCTGCTCCAATTGGTTCAGCTATTTCCAGAAACAATTGGGCTGTTACTTGTGAT 60
                ***** ** ** ** ** **    ** *****  * ***** ***** ** ***** **

wtgalox         AGTGCACAGTCGGGAAATGAATGCAACAAGGCCATTGATGGCAACAAGGATACCTTTTGG 120
optgalox        TCCGCTCAATCTGGTAATGAATGTAACAAGGCTATTGATGGAAACAAGGACACATTTTGG 120
                   ** ** ** ** ******** ******** ******** ******** ** ******

wtgalox         CACACATTCTATGGCGCCAACGGGGATCCAAAGCCCCCTCACACATACACGATTGACATG 180
optgalox        CATACTTTCTACGGTGCTAACGGAGATCCAAAACCACCTCACACTTATACAATTGACATG 180
                ** ** ***** ** ** ***** ******** ** ******** ** ** *********

wtgalox         AAGACAACTCAGAACGTCAACGGCTTGTCTATGCTGCCTCGACAGGATGGTAACCAAAAC 240
optgalox        AAGACTACACAGAACGTTAATGGTTTGTCTATGTTGCCTAGACAAGATGGAAACCAGAAT 240
                ***** ** ******** ** ** ********* ***** **** ***** ***** **

wtgalox         GGCTGGATCGGTCGCCATGAGGTTTATCTAAGCTCAGATGGCACAAACTGGGGCAGCCCT 300
optgalox        GGTTGGATCGGAAGACATGAAGTTTACTTGTCTTCCGATGGTACTAATTGGGGATCTCCT 300
                ** ********  * ***** *****  *    ** ***** ** ** *****    ***

wtgalox         GTTGCGTCAGGTAGTTGGTTCGCCGACTCTACTACAAAATACTCCAACTTTGAAACTCGC 360
optgalox        GTTGCTTCAGGTTCTTGGTTTGCTGACTCTACTACAAAGTACTCCAACTTCGAGACTAGA 360
                ***** ******  ****** ** ************** *********** ** *** *

wtgalox         CCTGCTCGCTATGTTCGTCTTGTCGCTATCACTGAAGCGAATGGCCAGCCTTGGACTAGC 420
optgalox        CCAGCTAGATATGTTAGATTGGTTGCTATTACAGAAGCTAATGGTCAACCTTGGACTTCT 420
                ** *** * ****** *  * ** ***** ** ***** ***** ** *********

wtgalox         ATTGCAGAGATCAACGTCTTCCAAGCTAGTTCTTACACAGCCCCCCAGCCTGGTCTTGGA 480
optgalox        ATTGCTGAGATCAACGTTTTTCAAGCTTCATCTTACACAGCTCCACAGCCTGGTTTGGGA 480
                ***** *********** ** ******   *********** ** ********* * ***

wtgalox         CGCTGGGGTCCGACTATTGACTTACCGATTGTTCCTGCGGCTGCAGCAATTGAACCGACA 540
optgalox        AGATGGGGTCCAACTATTGATTTGCCAATCGTTCCTGCTGCTGCTGCTATTGAGCCTACT 540
                 * ******** ******** ** ** ** ******** ***** ** ***** ** **

wtgalox         TCGGGACGAGTCCTTATGTGGTCTTCATATCGCAATGATGCATTTGGAGGATCCCCTGGT 600
optgalox        TCTGGAAGAGTTTTGATGTGGTCCTCATATAGAAATGACGCTTTTGGTGGATCCCCAGGT 600
                ** *** ****  * ******** ****** * ***** ** ***** ******** ***

wtgalox         GGTATCACTTTGACGTCTTCCTGGGATCCATCCACTGGTATTGTTTCCGACCGCACTGTG 660
optgalox        GGAATTACTTTGACATCTTCCTGGGATCCTTCAACTGGTATCGTTTCTGACAGAACTGTT 660
                ** ** ******** ************** ** ******** ***** *** * *****

wtgalox         ACAGTCACCAAGCATGATATGTTCTGCCCTGGTATCTCCATGGATGGTAACGGTCAGATC 720
optgalox        ACAGTTACTAAGCATGATATGTTCTGTCCAGGAATTTCTATGGACGGTAATGGACAAATC 720
                ***** ** ***************** ** ** ** ** ***** ***** ** ** ***

wtgalox         GTAGTCACAGGTGGCAACGATGCCAAGAAGACCAGTTTGTATGATTCATCTAGCGATAGC 780
optgalox        GTTGTTACAGGTGGAAACGATGCTAAGAAAACTTCATTGTACGATTCATCTTCCGACTCT 780
                ** ** ******** ******** ***** **    ***** *********  ***

wtgalox         TGGATCCCGGGACCTGACATGCAAGTGGCTCGTGGGTATCAGTCATCAGCTACCATGTCA 840
optgalox        TGGATTCCAGGTCCTGATATGCAAGTTGCTAGAGGATATCAGTCATCTGCTACAATGTCT 840
                ***** ** ** ***** ******** *** * ** *********** ***** *****

wtgalox         GACGGTCGTGTTTTTACCATTGGAGGCTCCTGGAGCGGTGGCGTATTTGAGAAGAATGGC 900
optgalox        GACGGTAGAGTTTTTACTATCGGTGGATCCTGGTCAGGTGGAGTTTTCGAAAAGAACGGA 900
                ****** * ******** ** ** ** ******   ***** ** ** ** ***** **

wtgalox         GAAGTCTATAGCCCATCTTCAAAGACATGGACGTCCCTACCCAATGCCAAGGTCAACCCA 960
optgalox        GAGGTTTACTCCCCATCCTCAAAGACATGGACTTCATTGCCAAATGCTAAAGTTAACCCT 960
                ** ** **   ****** ************** **  * ** ***** ** ** *****

wtgalox         ATGTTGACGGCTGACAAGCAAGGATTGTACCGTTCAGACAACCACGCGTGGCTCTTTGGA 1020
optgalox        ATGTTGACTGCTGATAAGCAAGGTTTGTATAGATCTGACAATCACGCTTGGTTGTTTGGT 1020
                ******** ***** ******** *****  * ** ***** ***** *** * *****

wtgalox         TGGAAGAAGGGTTCGGTGTTCCAAGCGGGACCTAGCACAGCCATGAACTGGTACTATACC 1080
optgalox        TGGAAGAAAGGATCAGTTTTCCAGGCTGGACCTTCTACAGCTATGAACTGGTACTATACT 1080
                ******** ** ** ** ***** ** ******   ***** *****************
```

```
wtgalox         AGTGGAAGTGGTGATGTGAAGTCAGCCGGAAAACGCCAGTCTAACCGTGGTGTAGCCCCT 1140
optgalox        TCTGGTTCCGGAGATGTTAAGTCCGCTGGTAAAAGACAATCAAATAGAGGAGTTGCTCCA 1140
                  ***    ** ***** ***** ** ** *** * ** ** **  * ** ** ** **

wtgalox         GATGCCATGTGCGGAAACGCTGTCATGTACGACGCCGTTAAAGGAAAGATCCTGACCTTT 1200
optgalox        GATGCTATGTGTGGTAACGCTGTTATGTACGACGCTGTTAAGGGAAAAATTTTGACTTTT 1200
                ***** ***** ** ******** *********** ***** ***** **  **** ***

wtgalox         GGCGGCTCCCCAGATTATCAAGACTCTGACGCCACAACCAACGCCCACATCATCACCCTC 1260
optgalox        GGTGGATCTCCTGACTATCAGGATTCCGACGCTACTACAAATGCTCATATTATCACTTTG 1260
                ** ** ** ** ** ***** ** ** ***** ** ** ** ** ** ** *****  *

wtgalox         GGTGAACCCGGAACATCTCCCAACACTGTCTTTGCTAGCAATGGGTTGTACTTTGCCCGA 1320
optgalox        GGTGAACCAGGAACATCCCCTAATACTGTTTTTGCTTCAAACGGTTTGTACTTTGCTAGA 1320
                ******** ******** ** ** ***** ******   ** ** ***********  **

wtgalox         ACGTTTCACACCTCTGTTGTTCTTCCAGACGGAAGCACGTTTATTACAGGAGGCCAACGA 1380
optgalox        ACATTCCACACTTCTGTTGTTTTGCCAGATGGTTCCACATTCATTACTGGTGGACAAAGA 1380
                ** ** ***** ********* * ***** **   *** ** ***** ** ** *** **

wtgalox         CGTGGAATTCCGTTCGAGGATTCAACCCCGGTATTTACACCTGAGATCTACGTCCCTGAA 1440
optgalox        AGAGGAATCCCTTTTGAGGATTCAACACCAGTTTTCACTCCTGAAATCTACGTTCCAGAG 1440
                 * ***** ** ** *********** ** ** ** ** ***** ******** ** **

wtgalox         CAAGACACTTTCTACAAGCAGAACCCCAACTCCATTGTTCGCGTCTACCATAGCATTTCC 1500
optgalox        CAAGACACTTTCTACAAGCAGAACCCTAATTCTATCGTTAGAGTTTACCATTCAATCTCT 1500
                ************************** ** ** ** *** * ** ******   ** **

wtgalox         CTTTTGTTACCTGATGGCAGGGTATTTAACGGTGGTGGTGGTCTTTGTGGCGATTGTACC 1560
optgalox        TTGTTGTTGCCAGATGGTAGAGTTTTCAACGGTGGAGGTGGATTGTGTGGAGATTGTACT 1560
                 * ***** ** ***** ** ** ** ******** *****  * ***** ********

wtgalox         ACGAATCATTTCGACGCGCAAATCTTTACGCCAAACTATCTTTACAATAGCAACGGCAAT 1620
optgalox        ACAAATCACTTTGACGCTCAAATTTTCACTCCAAACTACTTGTACAACTCCAATGGTAAC 1620
                ** ***** ** ***** ***** ** ** ********  * *****   *** ** **

wtgalox         CTCGCGACACGTCCCAAGATTACCAGAACCTCTACACAGAGCGTCAAGGTCGGTGGCAGA 1680
optgalox        TTGGCTACTAGACCTAAAATCACAAGAACTTCTACACAGTCCGTTAAGGTTGGTGGAAGA 1680
                 * ** **  * ** ** ** ** ***** *********  *** ***** ***** ***

wtgalox         ATTACAATCTCGACGGATTCTTCGATTAGCAAGGCGTCGTTGATTCGCTATGGTACAGCG 1740
optgalox        ATTACAATCTCTACTGATTCTTCCATTTCAAAGGCTTCTTTGATCAGATACGGTACTGCT 1740
                *********** ** ******** ***   ***** ** *****  * ** ***** **

wtgalox         ACACACACGGTTAATACTGACCAGCGCCGCATTCCCCTGACTCTGACAAACAATGGAGGA 1800
optgalox        ACACACACTGTTAATACTGACCAAAGAAGAATCCCATTGACATTGACTAACAATGGTGGA 1800
                ******** **************  *  * ** **  ****  **** ******** ***

wtgalox         AATAGCTATTCTTTCCAAGTTCCTAGCGACTCTGGTGTTGCTTTGCCTGGCTACTGGATG 1860
optgalox        AACTCCTACTCATTTCAGGTTCCATCTGATTCCGGTGTTGCTTTGCCTGGATATTGGATG 1860
                **   *** ** ** ** *****    ** ** ***************** ** ******

wtgalox         TTGTTCGTGATGAACTCGGCCGGTGTTCCTAGTGTGGCTTCGACGATTCGCGTTACTCAG 1920
optgalox        TTGTTCGTTATGAACTCTGCTGGTGTTCCTTCCGTTGCTTCAACAATTAGAGTTACTCAA 1920
                ******** ******** ** *********   ** ***** ** *** * ********

wtgalox         TGA 1923
optgalox        TAA 1923
                * *
```

Table S1. Oligonucleotide primer sequences (restriction sites used for cloning are in bold and underlined)

name	sequence
pPICZGalOx_fwd_HisTag	5'-ACTTTAAGAAGGA**GCGGCCGC**CATGGGCCATCATC-3'
pPICZGalOx_fwd_noHisTag_wt	5'-CATATCGAAG**GCGGCCGC**CATGGCCTCAGC-3'
pPICZGalOx_fwd_noHisTag_M1	5'-CATATCGAAG**GCGGCCGC**CATGGCCTCCGC-3'
pPICZGalOx_rev	5'-AGCCGGATC**TCTAGA**TCACTGAGTAACG-3'
pPIC3.5GalOx_fwd	5'-ACTTTAAGA**CCTAGG**GCCGCCATGGGCCATCATC-3'
pPIC3.5GalOx_fwd_noHisTag_wt	5'-CCATATC**CCTAGG**GCCGCCATGGCCTCAGCAC-3'
pPIC3.5GalOx_fwd_noHisTag_M1	5'-CCATATC**CCTAGG**GCCGCCATGGCCTCCGCAC-3'
pPIC3.5GalOx_rev	5'-AGCCGGA**GCGGCCGC**TCACTGAGTAACG-3'
pPICZGalOxopt_fwd	5'-ATGCATCT**GCGGCCGC**CATGGCTTCTG-3'
pPICZGalOxopt_rev_noHistag	5'-CGGGATCCG**TCTAGA**TTATTGAGTAACTC-3'
pPICZGalOxopt_rev_Histag	5'-CGGGATCCG**TCTAGA**TGAGTAACTC-3'
T7fwd	5'-AATACGACTCACTATAGGG-3'
T7rev	5'-GCTAGTTATTGCTCAGCGG-3'
5'AOX1	5'- GACTGGTTCCAATTGACAAGA-3'
3' AOX1	5'-GCAAATGGCATTCTGACATCC-3'
